# Genome Editing in iPSC-Based Neural Systems: From Disease Models to Future Therapeutic Strategies

**DOI:** 10.3389/fgeed.2021.630600

**Published:** 2021-03-15

**Authors:** Amy McTague, Giada Rossignoli, Arianna Ferrini, Serena Barral, Manju A. Kurian

**Affiliations:** ^1^Developmental Neurosciences, Great Ormond Street Institute of Child Health, University College London, London, United Kingdom; ^2^Department of Neurology, Great Ormond Street Hospital, London, United Kingdom

**Keywords:** induced pluripotent stem cells, CRISPR, gene editing, precision treatment, CRISPRi, CRISPRa, neurological disorders, disease modeling

## Abstract

Therapeutic advances for neurological disorders are challenging due to limited accessibility of the human central nervous system and incomplete understanding of disease mechanisms. Many neurological diseases lack precision treatments, leading to significant disease burden and poor outcome for affected patients. Induced pluripotent stem cell (iPSC) technology provides human neuronal cells that facilitate disease modeling and development of therapies. The use of genome editing, in particular CRISPR-Cas9 technology, has extended the potential of iPSCs, generating new models for a number of disorders, including Alzheimers and Parkinson Disease. Editing of iPSCs, in particular with CRISPR-Cas9, allows generation of isogenic pairs, which differ only in the disease-causing mutation and share the same genetic background, for assessment of phenotypic differences and downstream effects. Moreover, genome-wide CRISPR screens allow high-throughput interrogation for genetic modifiers in neuronal phenotypes, leading to discovery of novel pathways, and identification of new therapeutic targets. CRISPR-Cas9 has now evolved beyond altering gene expression. Indeed, fusion of a defective Cas9 (dCas9) nuclease with transcriptional repressors or activation domains allows down-regulation or activation of gene expression (CRISPR interference, CRISPRi; CRISPR activation, CRISPRa). These new tools will improve disease modeling and facilitate CRISPR and cell-based therapies, as seen for epilepsy and Duchenne muscular dystrophy. Genome engineering holds huge promise for the future understanding and treatment of neurological disorders, but there are numerous barriers to overcome. The synergy of iPSC-based model systems and gene editing will play a vital role in the route to precision medicine and the clinical translation of genome editing-based therapies.

## Introduction

Neurological disorders are the leading cause of disability and the second leading cause of death worldwide (Feigin et al., 2017). Despite apparent significant advances in investigation and treatment of these diseases, the health burden of neurological disorders has risen since 1990 (Feigin et al., 2019). In addition, the economic and societal impact of neurological disorders is substantial. As a result of the significant morbidity of many neurological conditions, the estimated annual cost of the nine commonest neurological disorders to society in the USA was $0.8 trillion (Gooch et al., 2017). A similar study in Europe found the cost of the 19 most prevalent brain and mental health disorders to be €798 billion per year (Olesen et al., 2012). Neurological disorders also include many rare diseases that are associated with considerable morbidity, societal, and economic impact (Graf von der Schulenburg and Frank, 2015). One of the reasons for this significant healthcare burden is that many neurological disorders still lack effective targeted treatments. The simultaneous development of two ground-breaking technologies, induced pluripotent stem cells (iPSCs) and genome engineering, has the potential to change this.

Development of effective treatments for neurological disorders is often challenging. The near inaccessibility of the human central nervous system hampers the investigation of underlying disease mechanisms and ascertainment of therapeutic targets. Animal models and heterologous cellular *in vitro* systems have provided insights into pathophysiological pathways of several neurological disorders, leading to advances in therapeutic approaches. Nevertheless, these models do not fully mimic human physiology, metabolism, and homeostasis, and only partially recapitulate the progression of the disease (Barral and Kurian, 2016). As a result, there is a higher failure rate of clinical trials and novel therapy development for neurological diseases (Kinch, 2015).

## Deriving Neuronal Systems From iPSC

With the advent of pluripotent stem cell-based technologies, both embryonic stem cells (ESCs) (Thomson, 1998) and induced pluripotent stem cells (iPSCs) (Takahashi et al., 2007), a new source of human neural cells became available. In particular, patient-derived iPSCs are enabling insight into rare neurological disorders for which no suitable models are available and are an ideal platform to test therapeutic approaches (Barral and Kurian, 2016; Mertens et al., 2016; Ghaffari et al., 2018; Kampmann, 2020; Silva and Haggarty, 2020). To date, several protocols have been published for the differentiation of pluripotent stem cells into neural cellular subpopulations, among which cortical, motor, dopaminergic, GABAergic, cholinergic, serotonergic neurons (Wada et al., 2009; Erceg et al., 2010; Wicklund et al., 2010; Kriks et al., 2011; Juopperi et al., 2012; Kirkeby et al., 2012; Carri et al., 2013; Espuny-Camacho et al., 2013; Liu et al., 2013; Maroof et al., 2013; Nicholas et al., 2013; Zhang et al., 2014; Du et al., 2015; Nizzardo et al., 2015; Hu et al., 2016; Lu et al., 2016; Nolbrant et al., 2017), and microglial cells, astrocytes and oligodendrocytes (Nistor et al., 2005; Ogawa et al., 2011; Emdad et al., 2012; Juopperi et al., 2012; Wang S. et al., 2013; Douvaras et al., 2014; Muffat et al., 2016; Abud et al., 2017; Tcw et al., 2017). The majority of these protocols are able to reproduce in a two-dimensional (2D) cellular model some of the processes observed in central nervous system (CNS) development and to derive neural populations that resemble those observed in the human fetal period. In order to overcome the lack of tissue-like complexity and the early stage fetal nature of the derived neural cells present in 2D derived neuronal systems, new protocols have been developed for the generation of three-dimensional (3D) brain organoids, which recapitulate some aspects of late gestational human brain (Lancaster et al., 2013; Pasca, 2018, 2019). Brain organoids not only allow complex neuronal network formation but can be maintained for long periods *in vitro* allowing analysis of neural circuit formation and complex physiological mechanisms which align more closely with human brain physiology in normal and disease states (Quadrato et al., 2017; Trujillo et al., 2019). However, maintaining cerebral organoids in long-term culture remains challenging due to oxygen, nutrient supply, and micro-environment issues, largely related to the inherent lack of vasculature. This was illustrated by intracerebral engraftment of human iPSC-derived cerebral organoids in mice, which led to vascularization, functional connectivity, and improved survival of organoids (Mansour et al., 2018). Giandomenico et al. used an air:liquid interface with sliced cerebral organoids to allow perfusion of the organoid core, improving neuronal survival, maturation, and axonal outgrowth and resulting in a functional circuit with mouse spinal cord explants in co-culture (Giandomenico et al., 2019, 2020). Other strategies to vascularize cerebral organoids have included ectopic expression of ETV2 (Cakir et al., 2019) or co-culture with iPSC-derived endothelial cells (Pham et al., 2018) or human umbilical vein endothelial cells (Shi et al., 2020). Advances in bioengineering and biomaterials have also sought to better reproduce the native extracellular matrix microenvironment. Although Matrigel and hydrogels are most commonly used, a floating scaffold consisting of synthetic microfilaments produced elongated embryoid bodies and improved cortical development in organoids(Lancaster et al., 2017). Bioreactors have also been developed to allow perfusion of nutrients and oxygen to organoid tissues (Lancaster et al., 2013; Qian et al., 2016). At the same time as improvements in organoid culture systems and subsequent to the development of whole-brain organoids, protocols using growth factor or small molecule regional patterning emerged, generating forebrain (Pasca et al., 2015; Birey et al., 2017; Quadrato et al., 2017; Sloan et al., 2018), midbrain-like (Jo et al., 2016; Qian et al., 2016), and hindbrain/cerebellar organoids (Muguruma, 2018) in addition to specific regional structures such as hypothalamic/ pituitary and retinal organoids (Parfitt et al., 2016; Kasai et al., 2020). These advances were followed by fusion of regional organoids to create subpallial-cortical fused organoids (Bagley et al., 2017; Birey et al., 2017; Xiang et al., 2017; Marton and Paşca, 2020), allowing the study of interactions between medial ganglionic eminence-derived GABAergic neurons and forebrain glutamatergic neurons. Most recently, cortico-striatal assembloids with functional neural circuits (Miura et al., 2020) and cortico-motor assembloids of hindbrain, spinal cord, and muscle have been generated (Andersen et al., 2020), demonstrating the potential of these models to investigate multi-organ aspects of neurological diseases.

**Figure 1:**
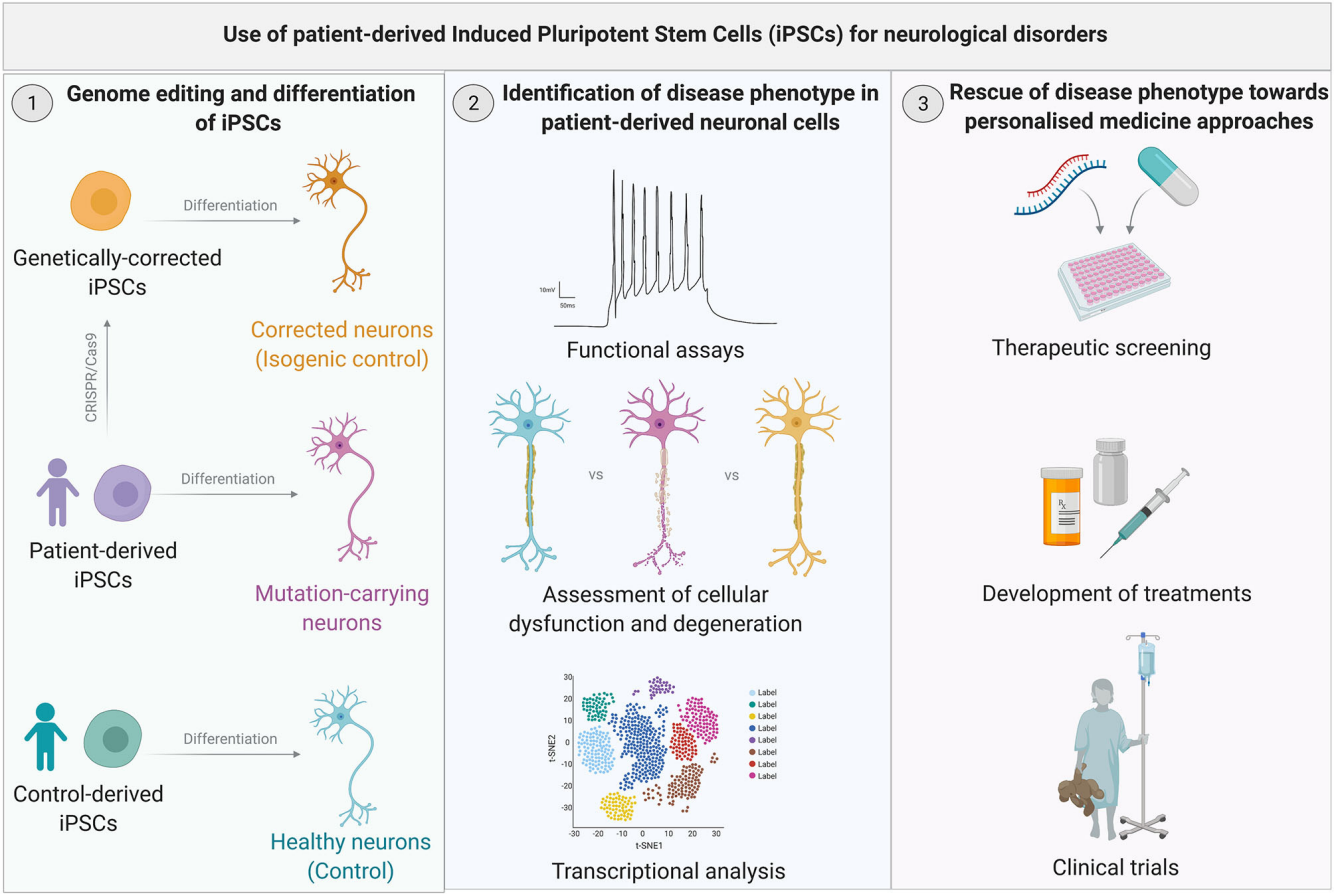
Use of patient-derived Induced Pluripotent Stem Cells (iPSC) for neurological disorders. **(Left)** iPSCs are readily derived from patients and controls through cellular reprogramming. Patient- and control-derived iPSCs are differentiated into the neuronal type relevant to the specific disease. Gene editing techniques such as CRISPR-Cas9 can be utilized to genetically correct the mutation in iPSCs to obtain isogenic control lines which only differ from the mutated iPSC lines by the genetic variant of interest. **(Middle)** Following genome editing and differentiation of iPSCs, derived cells are used to gain phenotypic insights into the specific disease mechanism through a variety of functional, morphological, and molecular analysis. **(Right)** Once a disease phenotype has been identified, iPSC-derived neuronal cells can be utilized for drug screens, which could accelerate personalized therapies for neurological disorders.

iPSCs patient-derived neurons, therefore, represent a unique *in vitro* model for the study of neurological disorders. They are an unlimited source of patient-derived cells, which retain the genetic background of the donor and allow investigation of disease-related mechanisms. Therefore, they provide a humanized model in which to test novel treatments, with the potential to accelerate the translation of novel therapeutic approaches. Moreover, they provide a renewable source of cells for cell replacement strategies to treat neurodegenerative disorders (Figure 1).

Since their inception, gene editing technologies have proved a useful tool in the development of *in vitro* disease models. Replacement of endogenous genomic sequences with exogenous donor DNA via homologous recombination (HR) and correct insertion of exogenous DNA at defined mammalian chromosomal locations was first developed in the 1980s (Smithies et al., 1984), and subsequently applied to the genomic modification of mouse embryonic stem cells (ESCs) (Hasty et al., 1991). The discovery of I-*Sce*I yeast meganuclease (Jacquier and Dujon, 1985), which promotes HR endogenous cellular mechanisms to repair DNA double-strand breaks (DSBs) in the presence of donor DNA, lead to the establishment of genome-editing strategies in murine cells (Choulika et al., 1995) and ESCs (Cohen-Tannoudji et al., 1998) based on proteins derived from unicellular organisms.

The advent of DNA-binding zinc-finger nuclease (ZFN) technology improved efficiency in genome-editing of mammalian cells (Bibikova et al., 2001), leading to the generation of the first knockout rat (Geurts et al., 2009). Following use in animals and cellular models (Petersen and Niemann, 2015), ZFNs-based genome editing was exploited for the correction of genetic mutations in patient-derived iPSCs (Soldner et al., 2011; Reinhardt et al., 2013; Kiskinis et al., 2018; Wang et al., 2018; Korecka et al., 2019), or for insertion of known disease-relevant mutations in iPSCs derived from healthy individuals (Verheyen et al., 2018), allowing direct investigation of specific genomic alterations and disease phenotypes. In addition, ZFNs were applied for the generation of engineered lines to study cell fate determination and improve iPSCs differentiation protocols (Hockemeyer et al., 2009), as well as to produce cell type-specific reporter systems for the investigation of disease pathogenesis (Zhang et al., 2016).

Genome editing technology further advanced with the advent of transcription activator-like effector nucleases (TALENs), which proved to be an efficient technology for the generation of animal models (Tesson et al., 2011). TALENs were further employed in the study of neurological disorders through the introduction of disease-causing mutations in control iPSCs (Wen et al., 2014; Lenzi et al., 2015; Akiyama et al., 2019) and/or correction of genetic mutations in patient-derived iPSCs (Maetzel et al., 2014; Wen et al., 2014; Li H. L. et al., 2015; Tanaka et al., 2018; Akiyama et al., 2019) leading to greater confidence in disease-underlying mechanisms and development of therapeutic approaches. Moreover, TALENs technology was used to develop reporter lines for stem cell-based research (Cerbini et al., 2015; Pei et al., 2015).

Rapidly following the development of TALENs technology, clustered regularly interspaced short palindromic repeats (CRISPR) with the CRISPR-associated protein (Cas9) system (Gasiunas et al., 2012; Jinek et al., 2012) demonstrated revolutionary potential to engineer the genome of mammalian cells in culture (Cong et al., 2013; Mali et al., 2013) and animal models (Wang H. et al., 2013). As for ZFNs or TALENs, CRISPR-Cas9 uses distinct DNA cleavage and binding modules. However, CRISPR-Cas9 system uses its own natural endonuclease and relies on a CRISPR RNA (crRNA) and a trans-activating RNA (transRNA) to specifically bind target DNA sequences and activating Cas9. Therefore, the long and complex process of engineered nuclease production was rapidly overcome by the plasticity and simplicity of generating different CRISPR-based approaches, which require only the design of a specific target-matching RNA. The extraordinary efficacy of CRISPR-Cas9, together with its great versatility for the generation of a broad range of substitutions, duplications, deletions, inversions, and many other complex alterations up to chromosomal rearrangements, have transformed the genome-editing field. There were, however, several limitations that required further improvements. Increased efficiency and reduction of off-target effects have been achieved through the engineering of Cas9 protein (Kleinstiver et al., 2015, 2016; Anders et al., 2016; Slaymaker et al., 2016; Chen et al., 2017; Casini et al., 2018; Hu et al., 2018; Lee et al., 2018; Nishimasu et al., 2018) and amendments to the design and structure of the guide RNA (Jinek et al., 2012; Hsu et al., 2013; Cui et al., 2018; Filippova et al., 2019; Moon et al., 2019), as well as the discovery and application of Cas proteins with different and specific gene-editing properties (Zetsche et al., 2015; Abudayyeh et al., 2016; Burstein et al., 2017). CRISPR-based technology has further developed to allow transcriptional inhibition (CRISPR interference, CRISPRi) or activation (CRISPR activation, CRISPRa). This CRISPR-based transcriptional modulation is achieved by repressor or activator transcription domains fused to a catalytically inactive Cas9 (dCas9) and guide RNAs directed to the promoter or regulatory regions of specific genes (Gilbert et al., 2013).

Owing to its robustness and flexibility, CRISPR-based gene editing systems have proven efficient for gene modification, gene expression regulation, epigenetic modulation, and chromatin manipulation, at both single-gene level and large-scale screening (Adli, 2018). Therefore, CRISPR-based technology has quickly become the preferred method of choice for genome-editing, particularly in iPSC model systems.

In this review, we will examine the role of genome engineering in iPSC-derived models of neurological disorders and discuss how the combination of these technologies has the potential to advance our understanding of disease mechanisms and to accelerate the translation of gene editing therapies.

### Creating New Disease Models for Neurological Disorders

**Figure 2:**
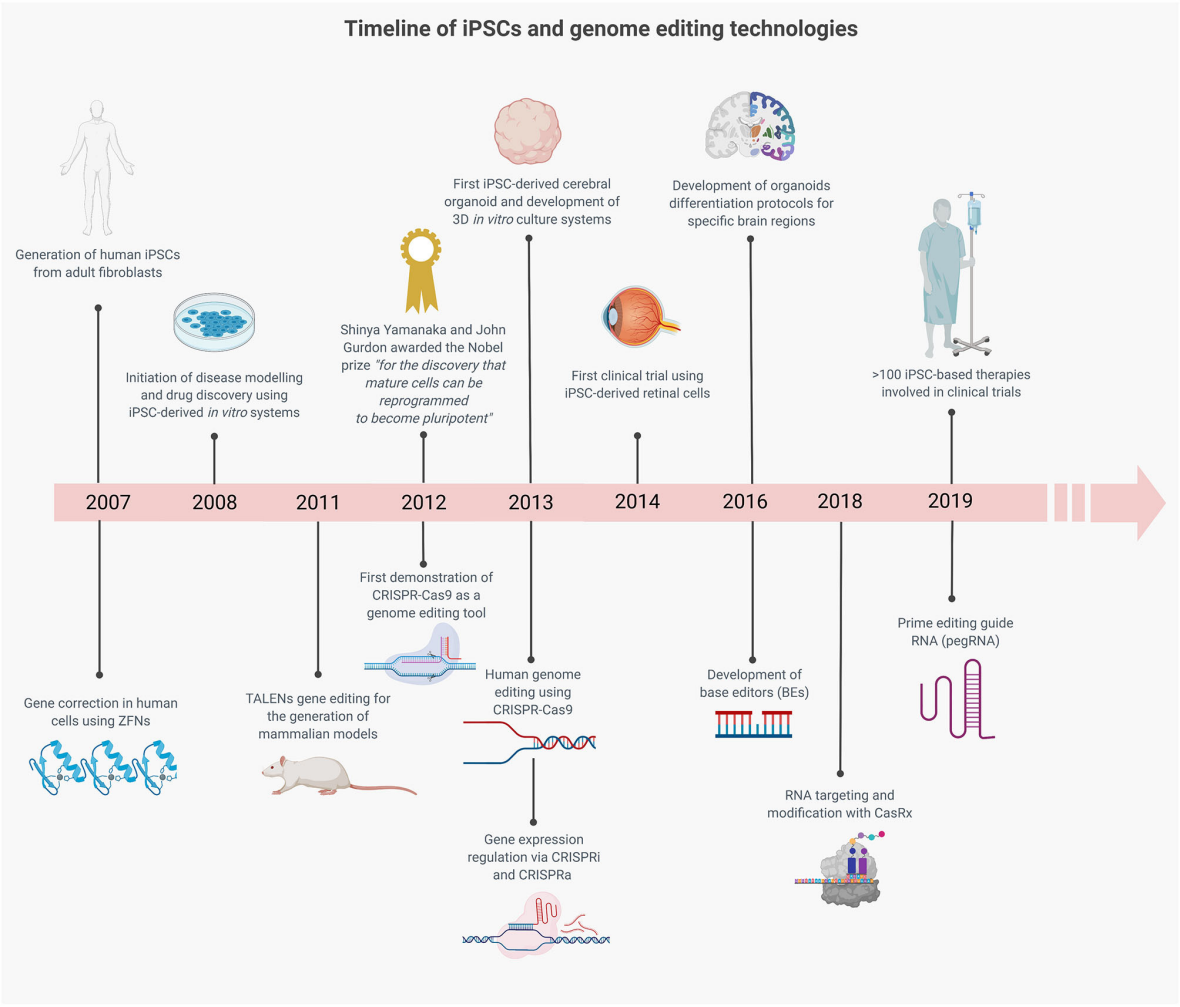
Timeline showing the parallel development of iPSC and gene editing technologies which have advanced *in vitro* modeling of neurological diseases. The combination of these technologies has the potential to advance our understanding of disease mechanisms and to accelerate the translation of gene editing therapies into the clinic.

The parallel development of iPSC and gene editing technologies has been serendipitous for advancing the understanding of neurological disorders (Figure 2) (Hockemeyer and Jaenisch, 2017). The ability to model neurological disorders in a humanized patient-derived model system has already led to many insights into disease mechanisms (Figure 1) (Barral and Kurian, 2016; Li et al., 2018), and this has been accelerated by synergy with genome editing. Initially TALENs and subsequently CRISPR-Cas9, have been utilized to create point mutations, insertion-deletions and to edit repeat expansions in iPSC lines from healthy control subjects (Supplementary Table 1). This has been particularly helpful in rare diseases where there is reduced accessibility to patient-derived samples for the generation of patient-derived iPSCs or where there is difficulty in obtaining patient samples with a particular genotype, such as *CHD8*-related autistic spectrum disorder (ASD) (Wang P. et al., 2017) and *KCNT1*-related epilepsy (Quraishi et al., 2019). Gene editing of patient-derived cells in rare neurological diseases such as spinal muscular atrophy led to phenotypic rescue with restoration of SMN expression, motoneuron survival, and neuromuscular junction formation and has paved the way for novel therapeutic approaches (Zhou et al., 2018). In Huntington's disease (HD), it has long been observed that CAG repeat length correlates with the age of disease onset. In human embryonic stem cells edited to contain differing CAG repeat lengths, cell-type specific phenotypic differences were seen which related to CAG length (Ooi et al., 2019). In addition, genome editing of control lines allows the study of many variants at once in the same genetic background, which may be more practical than collecting large numbers of patient lines. This paradigm has been elegantly used by Kwart et al., who generated a panel of 15 different familial Alzheimer disease (AD) variants in two genes *PSEN1* and *APP* to look for common disease mechanisms (Kwart et al., 2019). Using transcriptomic analysis, they were able to identify accumulation of βCTF mediating a common endosomal dysfunction (Supplementary Table 1).

### Isogenic Controls Reduce Variability and Improve Modeling

One of the strengths of the iPSC technology paradigm is the ability to model the effects of a variant in the native genetic milieu, which will naturally differ between individuals as part of normal genetic variation. Whilst iPSC systems offer an unparalleled opportunity to study and annotate human genetic variants associated with disease, this also represents a major shortcoming. Experimental variability due to reprogramming or differentiation differences between lines also make a contribution to system noise. In earlier iPSC studies, unrelated controls, often from in-house or stem cell bank lines or unaffected family members, were used for comparison with patient-derived mutant lines. However, extensive transcriptomic analyses from large stem cell repositories such as Human Induced Pluripotent Stem Cell Initiative (HipSci), European Bank for Induced pluripotent Stem Cells (EBiSC), and National Heart, Lung, and Blood Institute (NHBLI) have shown that variance is most greatly influenced by genetic differences between individuals (Germain and Testa, 2017) rather than between technical replicates, such as different clones from a single patient.

Therefore, for robust comparison of phenotypic effects, an isogenic line, which only differs by the genetic variant of interest, is now deemed necessary to ensure that observed differences are attributable to a specific genetic defect. The advent of gene editing technologies has revolutionized this aspect of iPSC modeling and has led to a great number of phenotypic insights (Supplementary Table 1). For example, early iPSC models of *SCN1A*-related epilepsies suggested conflicting disease mechanisms, with some studies finding increased sodium current in excitatory neurons contradicting the loss of function noted in GABAergic neurons in animals and other iPSC studies. Liu et al. used TALENs to correct an *SCN1A* variant from a patient-derived line and CRISPR-Cas9 to knock in a fluorescent reporter expressed in GABAergic neurons (Liu et al., 2016). By comparison with isogenic controls, they were able to show a selective loss of function in GABAergic neurons, which was later confirmed in another study (Sun et al., 2016). Subtler phenotypes in later-onset disorders such as Parkinson's disease (PD) have also been revealed by correction of disease-causing variants; Reinhardt et al. demonstrated that cellular phenotypes such as neurite outgrowth, abnormal autophagy, and tau and α-synuclein accumulation were *LRRK2*-dependent in familial PD (Reinhardt et al., 2013). Isogenic controls also have the power to show that some cellular phenotypes are dependent on genetic background even in monogenetic conditions; correction of *LRRK2* variants in another study exposed both *LRRK2*-dependent and *LRRK2*-independent effects, which were likely related to genetic background and reflected the heterogeneous clinical features and variable severity in familial PD (Nickels et al., 2019). This enabled the identification of serine metabolism as a putative genetic modifier of familial PD. Isogenic controls are therefore essential in both monogenic highly penetrant disorders and disorders where there is phenotypic variability. For example, in neurological diseases caused by triplet repeat expansions such as Friedrich ataxia (FA) there is significant clinical heterogeneity. This can be due to both gene-dependent factors such as expansion size and gene-independent factors such as genetic background and environmental factors (Schreiber et al., 2019). Therefore, isogenic controls were necessary to discriminate truly *FRDA*-intrinsic effects, with restoration of frataxin expression and other FA phenotypic features in response to ZFN excision of the expanded GAA repeat (Li Y. et al., 2015).

### Gene Editing to Investigate Complex Disorders

It would be expected that genetic modifiers make an even larger contribution to complex diseases, along with environmental and non-genetic risk factors such as lifestyle. Genome-wide association studies (GWAS) identify risk loci which are statistically associated with the risk of developing complex disorders such as sporadic PD or AD, but the single nucleotide polymorphisms or alleles identified usually have individually small effect sizes, making detection of a subtle phenotype in an iPSC system challenging. Gene editing can answer some of these challenges. Differing genotypes of the apolipoprotein E4 gene *APOE4* are associated with risk of sporadic AD (Raman et al., 2020). iPSCs from healthy subjects were converted from neutral risk (APOE3) to APOE4 (high risk) by gene editing, and patient-derived iPSCs were conversely converted from APOE4 to APOE3 in several studies (Lin et al., 2018; Meyer et al., 2019). This “rescue” of the risk status of iPSCs derived from patients with the propensity to develop AD later in life led to a reversal of AD phenotypes such as the inability of glial cells to clear extracellular Aβ and increased Aβ aggregates in cerebral organoids. In addition, accelerated neuronal differentiation and reduced renewal of neural progenitors were reversed by gene editing (Meyer et al., 2019). This study also highlighted that iPSC systems could be highly useful to investigate late-onset disorders, which may seem counter-intuitive as iPSC-derived neurons are considered immature, representing a fetal neuronal development stage even after several months in culture (Vera and Studer, 2015). Nevertheless, iPSC systems represent an opportunity to model early neurodevelopment and therefore may detect early disease features which are pre-symptomatic, such as the APOE4-dependent altered neuronal differentiation seen in the sporadic AD model (Meyer et al., 2019), which could result in neuronal vulnerability to other stressors and environmental factors.

Gene editing can also be used to investigate the cellular effects of risk alleles in models of sporadic disease. Schrode et al. used CRISPR-Cas9 to introduce a known risk allele into control iPSC lines and CRISPRi/a to modulate the top-ranked schizophrenia-associated expressive quantitative trait loci (eQTL) (Schrode et al., 2019), including *SNAP91, TSNARE1*, and *CLCN3*. An eQTL is a genomic region or single nucleotide polymorphism (SNP) associated with the expression of a gene, in this case, identified from post-mortem (PM) gene expression studies in schizophrenia patients. Extensive phenotypic analyses of neurons differentiated from isogenic pairs showed that although there were gene-dependent effects, these converged on a common synaptic defect. There was a particular phenotypic overlap between the effects of *SNAP91* CRISPRi and *TSNARE1* CRISPRa. In addition, this study showed that the effect sizes seen in the iPSC-derived neurons and organoids were larger than those in PM studies. Moreover, the combinatorial perturbation of genes rather than an additive model better approximated gene expression signatures in schizophrenia datasets and revealed enrichment in gene sets involved in secretion of glutamate and other neurotransmitters, synaptic vesicle trafficking, and a postsynaptic glutamate receptor pathway. This demonstration of a complex synergistic impact of common variants on cellular and molecular phenotypes for schizophrenia emphasizes the importance of considering this particular disorder's polygenic nature and may apply more broadly to many complex non-Mendelian genetic disorders. Potentially, *in vitro* systems such as iPSC models could be used to identify novel eQTLs such as SNPs, which modify gene expression in different iPSC-derived cell lineages, and which can then be directly investigated for their functional effect (Soldner and Jaenisch, 2019).

### Interrogating the Transcriptome and Methylome With Genome Editing

Genome-wide analysis of gene expression in iPSC models using RNA sequencing has been made possible by the generation of appropriate isogenic controls and has led to many mechanistic insights (Supplementary Table 1). *CHD8* has been found to be mutated in both ASD and schizophrenia. To understand the downstream effects of *CHD8* variants and identify common pathways, Wang et al. generated *CHD8* knockout lines using gene editing (Wang P. et al., 2017). RNAseq revealed *CHD8* regulates known ASD genes *TCF4* and *AUTS2* and affects GABAergic interneuron development by modulating *DLX* gene expression. Pathway analysis of differentially expressed genes (DEGs) was enriched for genes involved in the Wnt/β-catenin pathway, representing a potential drug target.

Gene editing in iPSC systems has been instrumental in understanding pathophysiological mechanisms related to the modification of a disease-associated gene. However, genome engineering can be used in combination with transcriptomic analysis to better investigate underlying disease mechanisms. To investigate early-onset AD in patients with Down syndrome, CRISPR-Cas9 was used to delete the supernumerary copy of *APP* in T21 lines and inducible CRISPRa to upregulate *APP* gene expression (Ovchinnikov et al., 2018). *APP* gene dosage was shown to be connected to β-amyloid production but not to other AD cellular phenotypes, including apoptosis. The use of CRISPR screens to delineate disease mechanisms is discussed further below.

In addition to DNA editing, it is possible to alter DNA methylation states using similar genome engineering approaches. Proof of principle was established with gene editing in Fragile X syndrome, a common inherited form of intellectual disability caused by expansion of a CGG repeat in the *FMR1* gene. CRISPR-Cas9 editing of the repeats revealed their role in demethylation of the upstream CpG island of the *FMR1* promoter, resulting in an open chromatin state and initiation of transcription with FMR1 levels restored in derived neurons (Park et al., 2015). A further study used a catalytically inactive Cas9 (dCas9) fused to a DNA methyltransferase domain (dCas9-Tet1) to directly demethylate the CGG repeat, rescuing FMR1 expression (Liu et al., 2018). A similar approach was taken by Kantor et al. to demethylate *SNCA* in a PD model, rescuing SNCA levels and mitophagy defects in patient-derived dopaminergic neurons without altering the rest of the methylome (Kantor et al., 2018).

### Modeling Neurodevelopmental Impact, Cell-Type Vulnerability, and Multi-System Diseases

A further advantage of genome engineering in iPSC systems is the ability to assess the impact of gene dosage and genetic variation at differing neurodevelopmental time-points. In tuberous sclerosis, heterozygous variants in *TSC1* or *TSC2* (mTOR pathway genes) cause a multisystem disorder including epilepsy, developmental delay and propensity to non-malignant overgrowth such as cortical tubers. Martin et al. examined the impact of tuberous sclerosis variants on neuronal precursors, noting that MTOR inhibitors such as rapamycin did not restore abnormal proliferation and neurite outgrowth to the same degree as gene correction, indicating mTOR-independent early disease mechanisms that have important implications for future treatments (Martin et al., 2020). Previous studies have identified biallelic *TSC1/2* mutations in cortical tubers, suggesting a second hit or somatic mutation may be required (Winden et al., 2019). Indeed TALEN or CRISPR-engineered biallelic patient lines showed an effect of gene dosage on mTOR activation and reduced synaptic activity (Sundberg et al., 2018; Winden et al., 2019). Blair et al. edited a control iPSC line to create a *TSC2* loss of function variant with an inducible loss of function variant in the other allele (Blair et al., 2018). During expansion of neural progenitors, mosaic biallelic inactivation was shown to lead to formation of dysplastic cells, supporting the two-hit hypothesis. Indeed, inducible gene editing in cerebral organoids could provide a viable future platform to investigate disease mechanisms in other similar disorders where cerebral somatic mosaicism is implicated, such as malformations of cortical development (Poduri et al., 2012; Verheijen, 2019).

iPSC modeling is ideal for disorders such as tuberous sclerosis due to the early onset of clinical disease and the ability to generate relevant neuronal types of any lineage, such as cerebellar Purkinje cells or neural crest cells (Sundberg et al., 2018; Delaney et al., 2020). Cell-type vulnerability can also be investigated with iPSC models and gene editing. In AD, some brain regions appear to be more severely affected by Aβ plaque deposition. Neuronal differentiation was directed to either to caudal (hindbrain) or rostral (forebrain) fates from patient-derived iPSCs bearing *APP* mutation and showed more severe tau responses in forebrain neurons, confirming brain-region susceptibility in AD (Muratore et al., 2017). Investigation of the impact of the *APOE4* genotype in microglia also revealed relative cell-type vulnerability for sporadic vs. familial AD (Konttinen et al., 2019).

Beyond the central nervous system, the ability to generate multiple lineages is helpful for multi-system disorders. In *SCN1A*-Dravet syndrome, increased risk of SUDEP may relate to cardiac sodium channel dysfunction (Frasier et al., 2018). Cardiomyocytes were generated from patient-derived iPSCs; mutant cardiomyocytes showed increased sodium current density and spontaneous contraction rates, partly explained by a compensatory increase in *SCN5A* expression. In Nieman-Pick disease, both neurons and hepatocytes were differentiated from patient-derived iPSCs with biallelic mutations in *NPC1* (Maetzel et al., 2014). Phenotypic rescue of the autophagy defect was demonstrated by TALEN gene correction in both cell lineages with implications for future therapy. In FA, cardiomyopathy is a major cause of morbidity and mortality. ZFN editing of patient-derived iPSC and control lines to create isogenic controls showed rescue of frataxin expression, lipid accumulation, and a hypertrophy-specific transcriptomic signature (Li J. et al., 2019). Most recently, iPSCs from FA patients were differentiated into dorsal root ganglia organoids, generating sensory neurons, which were co-cultured with intrafusal muscle fibers, recapitulating the human proprioceptive network (Mazzara et al., 2020). This enabled testing of two different CRISPR therapeutic approaches, with improved phenotypic rescue following excision of the entire first intron rather than the expanded GAA tract alone.

Exploiting the pluripotency of iPSCs can also reveal tissue-specific putative disease mechanisms and treatment approaches. Oikari et al. investigated the impact of familial AD mutations in *PSEN1* on blood brain barrier (BBB) formation by differentiating induced brain endothelial cells (iBECs) from patient-derived and isogenic lines (Oikari et al., 2020). Mutant iBECs showed abnormal tight and adherin junction protein expression. Focused ultrasound is a novel approach to open the BBB. In iBEC cultures, AD and isogenic iBECs responded differently, suggesting this may be a novel approach to improve CNS drug delivery in AD.

### Combining Genome Editing With Environmental Perturbation

It is also possible to combine iPSC systems and genome engineering to study the additional effect of environmental factors or stressors. Both mutations in the tau gene, *MAPT*, and exposure to environmental toxins such as heavy metals are known to increase the risk of developing progressive supranuclear palsy, a neurodegenerative disorder characterized by the accumulation of tau aggregates. Induced neurons differentiated from a patient-derived *MAPT* iPSC line and the corresponding gene-corrected isogenic control were exposed to heavy metals, including chromium and nickel (Alquezar et al., 2020). Heavy metals increased tau levels and induced apoptosis in all cell lines, but *MAPT* mutant lines showed increased vulnerability, indicating a link between genetic predisposition and environmental factors. Environmental factors in the form of temperature can worsen some genetic conditions. Pathogenic variants in *ATP1A3* cause a range of neurological conditions, including alternating hemiplegia of childhood, where attacks of hemiplegia can be triggered by stressors such as illness or change in temperature. Patient-derived neurons displayed hyperactivity on microelectrode array analysis following temperature elevation compared to gene-corrected neurons (Snow et al., 2020). Similarly, ultra-violet irradiation of iPSC-derived neuronal cultures from a patient with *ERCC6*-related Cockayne syndrome recapitulated the DNA damage and premature aging seen *in vivo*, which were rescued in the gene-corrected lines (Wang et al., 2020).

## Crispr-Cas9 Based Screening for Neurological Disorders

### CRISPR Knock-Out and Knock-In Screening for Disease-Specific Investigation

**Figure 3:**
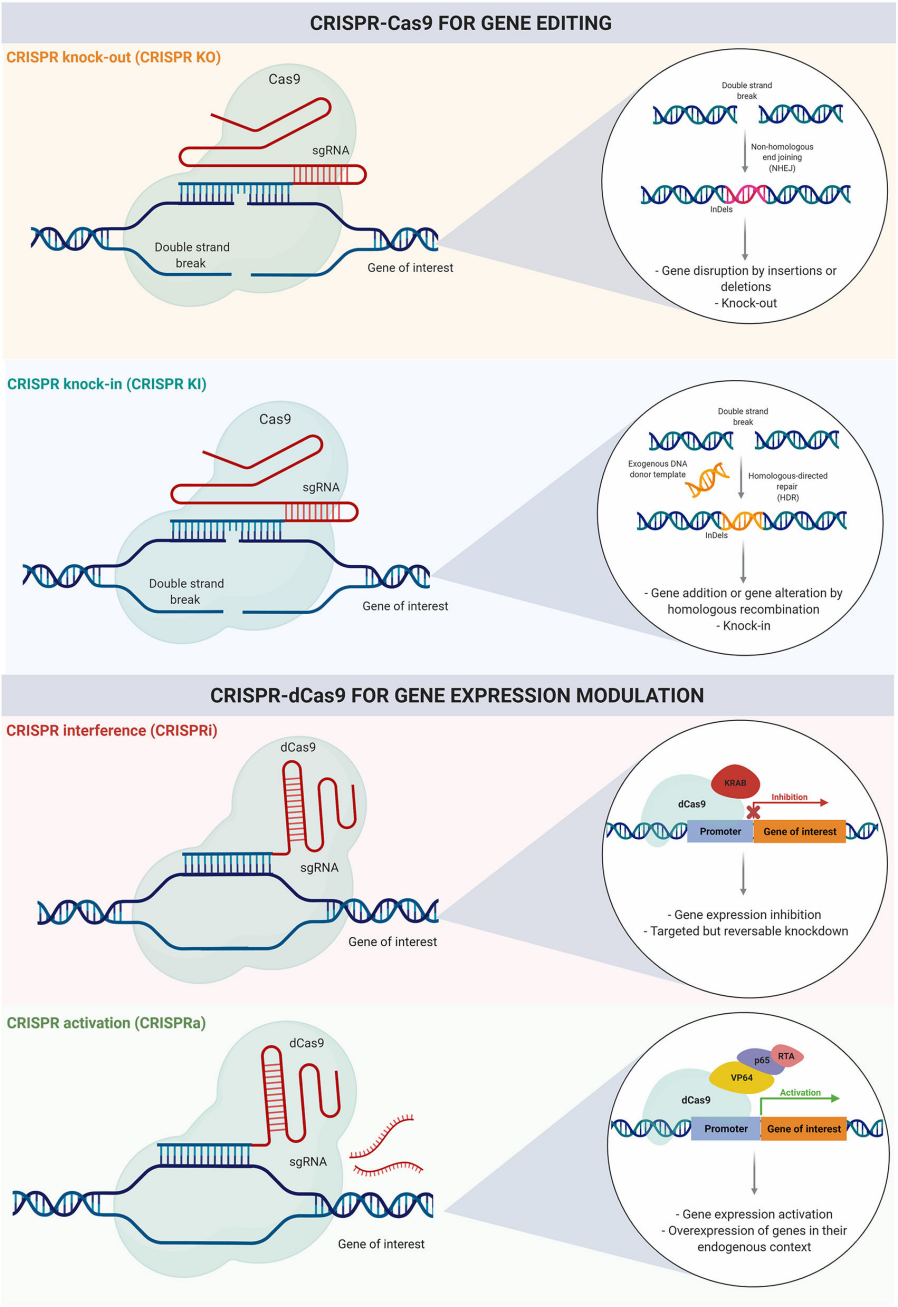
CRISPR-Cas9 for gene editing and gene expression modulation. Genome engineering using CRISPR-Cas9 can be used for gene editing with insertions or deletions resulting in gene knock-out, or for knock-in using homologous recombination. Recently, CRISPR has evolved toward gene expression modulation; CRISPR intereference (CRISPRi) and CRISPR activation (CRISPRa) can be used for transient inhibition or activation of gene expression respectively.

In addition to numerous applications in single-gene engineering, CRISPR-Cas9 has evolved into a functional genomics tool. It has been adapted to target multiple genes simultaneously, giving rise to several genome-wide CRISPR knock-out (CRISPR KO) and knock-in (CRISPR KI) studies (Cong et al., 2013). CRISPR-Cas9 based screening combined with high throughput methods for functional evaluation is a powerful approach to systematically elucidate gene function in health and disease states, with further implications for diagnosis and treatment development (Figure 3).

Genome-wide CRISPR-Cas9 screens were initially employed in dividing immortalized cell-lines, allowing the reliable introduction of variants at known sites in the genome, facilitating selection of clones, and easy scaling-up of functional evaluation. This “forwards genetics” approach allowed unbiased phenotypic screening and identified genetic determinants of diptheria infection and melanoma treatment resistance, amongst other examples (Shalem et al., 2014; Wang et al., 2014). However, heterologous cellular models lack relevant cellular pathophysiological phenotypes, in particular for neurological disorders. Therefore, patient iPSC-derived neuronal cells have been employed as a new *in vitro* model for secondary validation of identified hits. Potting and colleagues (Potting et al., 2017) performed a selection screen in HEK293 cells expressing endogenous PARKIN, an E3 ligase that promotes mitophagy by ubiquitinating mitochondrial proteins and whose mutations cause familial PD. Among 53 identified positive and negative regulators of PARKIN expression, a transcriptional repressor network including THAP11 was further validated in iPSCs-derived excitatory neurons. Heterozygous KO of *THAP11* significantly increased *PARK2* mRNA and PARKIN protein levels and altered mitochondrial ubiquitinylation in neurons. This analysis demonstrated that PARKIN levels affect mitophagy in the early damage-induced state, further elucidating the role of mitophagy in PD's pathogenesis.

Similarly, a CRISPR KO screen was performed to understand underlying mechanisms and therapeutic targets for treatment of frontotemporal dementia and amyotrophic lateral sclerosis (c9FTD/ALS) (Kramer et al., 2018). Both neurological conditions are characterized by hexanucleotide repeat expansions in *C9orf72* gene, which translate into dipeptide repeat (DPR) aggregation-prone proteins. CRISPR-Cas9 screening was initially performed in myelogenous leukemia cells to identify suppressors and enhancers of C9orf72 DPR-mediated toxicity. A second validation was then performed in primary mouse neurons, and the effect of a final hit was unraveled in motor neurons derived from C9orf72-ALS iPSCs. This approach identified endoplasmic reticulum (ER) function and stress as important mechanisms involved in c9FTD/ALS pathogenesis. In addition, it allowed the identification of novel mitigators of DPR-induced toxicity, such as down-regulation of the ER-resident protein TMX2, which improved ALS-derived motor neurons survival and could represent a potential therapeutic target. Another CRISPR KO screen was performed in retinal pigment epithelium cells with validation in patient-derived iPSCs and differentiated motor neurons to discover genetic modifiers of C9ORF72 DPR production (Cheng et al., 2019). Among enriched genes involved in RNA nuclear export and translation, DDX3X, an RNA helicase, was identified to directly bind the repeat expansions in *C9orf72* RNA, repressing the translation of DPR proteins. Decreasing DDX3X increased endogenous DPRs in patients' iPSCs, while exogenous expression of DDX3X reduces DPR levels and rescues nucleocytoplasmic transport abnormalities, and improved survival of iPSC-differentiated motor neurons. This work indicate that strategies to increase DDX3X expression or activity may have therapeutic potential for c9FTD/ALS.

To date, very few genome-wide CRISPR KO and CRISPR KI screens have been undertaken for the study of neurological disorders, and just a small fraction was directly performed on iPSC-derived neurons. This is likely a consequence of the considerable resources and investments in time that iPSC-based systems require. This issue has been addressed with two approaches: (a) perform genome-wide screening in iPSCs or iPSCs-derived cellular subtypes generated from healthy individuals, which can be followed by hit validation in a disease model; (b) perform a focused analysis on a wide set of genes or mutations known to confer susceptibility to a particular disease.

The first approach is exemplified in a genome-wide screen to identify host factors required for Zika virus (ZIKV) infection, which was performed in healthy iPSC-derived neuronal progenitors (NPs) (Li Y. et al., 2019). ZIKV infection during early gestation results in reduced differentiation of NPs and cell death during early cortical development with consequent fetal brain abnormalities (Mlakar et al., 2016; Tang et al., 2016). A genome-wide CRISPR KO screen using a pooled library of 187,535 sgRNAs identified host factors that confer resistance to ZIKV-induced cell death, which were mainly associated with heparan sulfation, endocytosis, and interferon pathways. Pharmacological modulation of the identified pathways showed a protective effect against ZIKV infection in NPs. Among negative regulators of the interferon pathway, the screen identified *ISG15*, in which mutations have been linked to mycobacterial susceptibility (Bogunovic et al., 2012). The knockout of *ISG15* protected iPSC-derived cerebral organoids from ZIKV infection in contrast to control cerebral organoids, consistent with a critical role for the interferon pathway in ZIKV infection. The comprehensive screen performed on iPSC-derived NPs identified unknown neuronal-specific pathways and specific genes involved in ZIKV infection, not previously highlighted in genome-wide screenings performed on other cellular models (Savidis et al., 2016). These results underly the importance of using biologically relevant cellular models for functional investigations.

On the other side, finding common functional pathways between a specific set of genes or mutations known to confer susceptibility to a particular disorder can be useful for elucidating pathophysiological mechanisms of disease and to identify possible shared therapeutic targets, limiting the resources that a genome-wide iPSC-based screen requires. For example, Deneault and colleagues performed a CRISPR KO of 10 relevant genes that confer susceptibility to ASD (Deneault et al., 2018) (Supplementary Table 1). Despite gene-dependent phenotypes, transcriptomic analysis revealed converging effects with a reduction in synaptic activity in excitatory neurons derived from CRISPR KOs vs. control line. Although ASD susceptibility genes belong to different pathways, isogenic iPSC lines revealed disruption of common signaling networks associated with neuron projection and synapse assembly and a shared cellular phenotype, characterized by reduced functional connectivity of excitatory neurons. Another study focused instead on familial AD reported a CRISPR-Cas9 based screening of 200 heterozygous disease-causing mutations in amyloid precursor protein (APP) and presenilin isoforms (PSEN1 and PSEN2) (Kwart et al., 2019) (Supplementary Table 1). Transcriptomic and translatomic analyses in cortical neurons derived from the panel of genome-engineered iPSC lines showed that familial AD mutations in the two different genes have overlapping effects on endocytic/endosomal trafficking-associated pathways, previously associated with late-onset AD. This result suggests that a shared network of cellular and molecular changes may underlie both familial and sporadic AD pathogenesis, representing a common therapeutic target.

CRISPR KO and CRISPR KI screening technology together with iPSC-based disease modeling can therefore improve our understanding of pathophysiological mechanisms and potentially guide therapeutic interventions for neurological disorders.

### CRISPRi/a Screening to Understand Human Neurological Biology and Disease States

CRISPRi/a tools allow the modulation of gene expression at the endogenous transcription level, an advantage compared to RNA interference (RNAi) and complementary DNA (cDNA) library screening approaches (Larson et al., 2013; Konermann et al., 2015).

CRISPRa ability to induce robust expression at specific targets provides a transformative tool that has been already tested for the stimulation of neuronal differentiation. Using a mixed pool of gRNAs directed against either *NGN2* or *NEUROD1*, Chavez et al. were able to achieve a rapid and robust differentiation of iPSCs into a neuronal phenotype (Chavez et al., 2015). Further efforts in the improvement of CRISPRa application for iPSCs differentiation into neurons and astrocytes focused on the development of a single all-in-one vector that contained CRISPR components and modular cassettes for simultaneous and stable expression of several sgRNAs, achieving a higher and precise activation of multiple transcription factors than the one-by-one vectors that relied on co-transfection (Li et al., 2017). Moreover, the implementation of CRISPRa-based photoactivatable transcription systems enabled high inducible activation of endogenous target genes in various human cell lines and demonstrated to induce *NEUROD1* upregulation and neuronal differentiation from iPSCs (Nihongaki et al., 2017).

CRISPRi-based platforms have been used for the interrogation of gene function in iPSC-derived glutamatergic neurons (Tian et al., 2019). Healthy control subject-derived iPSCs have been engineered for stable expression of both inducible Neurogenin 2 (Ngn2), allowing large-scale production of glutamatergic neurons, and inducible dCas9-KRAB, to silence target gene expression. The use of this CRISPRi strategy and downstream functional readouts highlighted genes essential for neuronal survival, specific neuronal functions of ubiquitous genes, and genes involved in maintenance of neuronal morphology. Black et al., conversely, developed a CRISPRa screening approach to profile the contribution of putative human transcription factors to neuronal cell fate specification (Black et al., 2020). Using single and paired pooled screens, they identified several proneural factors characterized by a range of conversion efficiencies, from a clear neurogenic activity to the requirement of the co-expression with other factors to obtain a complete differentiation. Interestingly, they identified sets of transcription factors that improved neuronal differentiation efficiency, maturation, and subtype specification. These studies illustrate the utility of CRISPR-based modulation screening to systematically detect context-specific roles of human genes, providing a deeper mechanistic understanding of neuronal physiology and development, and supporting the use of unbiased methods to reach a more comprehensive knowledge of these processes.

CRISPRi/a have also become attractive tools for parallel in-depth molecular analyses of multiple disease states. The two technologies have been used to evaluate the feasibility of precise modulation of the expression of critical neurodegenerative disease-related genes (Heman-Ackah et al., 2016). Links between altered expression of pathogenic variants affecting *SNCA, MAPT, HTT*, and *APP* are well established to PD, FTD, AD, and HD, respectively, which share the common molecular events of protein misfolding, aggregation, and accumulation, collectively known as proteinopathies. Overlap of clinical features is often observed between synucleinopathies and other neurodegenerative diseases. The first in parallel CRISPRi-mediated modulation of all aforementioned genes was achieved in the same cell using a combination of sgRNAs, demonstrating the feasibility of performing complex manipulations of gene expression profiles to probe the contributions of specific genes and combinations thereof to disease phenotypes. This study demonstrated the possibility of using CRISPRi-based screening to understand the contribution and possible interplay of different disease-related genes in a multiplex setting. Moreover, the study confirmed the possibility to precisely modulate *APP* using CRISPRa in iPSC-derived neurons, which could be used to mimic later-onset degenerative phenotypes, often missing in iPSC-derived neuronal cultures. This would provide more realistic modeling of neurodegenerative disorders, avoiding chemical aging, external stressors, or direct administration/over-expression of disease-related proteins.

The simultaneous modulation of expression of different genes through CRISPRi/a gives the possibility to unravel the contribution of common risk variants to complex genetic disorders. Schrode et al. (2019) applied CRISPRi/a and isogenic strategies to manipulate and evaluate endogenous gene expression of schizophrenia-associated common variants as discussed in the disease modeling section (Supplementary Table 1). Therefore, the application of CRISPRi/a to iPSC models of neurological disorders can further extend the utility of both systems for the understanding of pathophysiological mechanisms of disease and may aid in the development of precision therapies and novel CRISPR-Cas9-based therapeutic approaches.

## Recent Advances in Gene Editing for Neurological iPSC-Modeling and Future Therapies

CRISPR-based engineering technologies have enabled researchers to dissect the function of specific genetic elements or correct disease-causing mutations. In parallel, CRISPR tools are now being implemented for active control and modulation of desired messenger RNAs (mRNAs). This allows the interrogation of transcriptome dynamics and the establishment of causal links between observed transcriptional changes and cellular phenotypes. Previously, RNA interference (RNAi) technology enabled inhibition of desired transcripts using micro RNAs (miRNAs), but this carried significant off-target effects due to cross-reaction with targets of limited sequence similarity and mis-targeting effects linked to endogenous miRNAs (Flynt and Lai, 2008). Investigation of Cas proteins able to target RNA led to the development of an engineered RNA-guided and RNA-targeting enzyme (CasRx) (Konermann et al., 2018), which showed improved efficiency in knocking down endogenous mRNA levels compared to RNAi technology, allowing ready manipulation of alternative splicing in human cells. Moreover, Konermann and colleagues successfully applied Cas-Rx editing in a patient-derived cortical neuronal model of FTD to modulate the balance of tau isoforms. Some forms of FTD with parkinsonism linked to chromosome 17 (FTDP-17) and other tauopathies are caused by mutations in the intron following exon 10 of *MAPT* (Boeve and Hutton, 2008). These variants disrupt an intronic splicing site and increase expression of the 4R tau isoform that contains more microtubule-binding domains (Kar et al., 2005), inducing pathological changes and driving the progression of neurodegeneration (Schoch et al., 2016). CasRx-mediated exon exclusion reduced 4R tau expression to a level similar to unaffected control neurons, suggesting that this technology can be exploited for transcriptional modulation in *in vitro* models. Interestingly, the small size of CasRx was amenable to packaging in adeno-associated virus (AAV) for delivery into post-mitotic neurons, encouraging future clinical applications in treatment of neurological disorders, and could be paired with an array encoding multiple guide RNAs for multiplexing. Therefore, CasRx technology paves the way for transcriptome engineering and RNA-targeting therapeutic applications.

To date, most functional genomic studies have focused on protein-coding genes. The increased understanding of the role of non-coding genome sequences in cellular processes, normal development, and disease states has promoted the interrogation of this largely unexplored domain. CRISPR-based editing can be a useful approach to enable mechanistic studies of specific non-coding genome functions and complex biology of the whole genome. Whole-genome screening technology has been applied in iPSCs and other cell types to interrogate functional contributions of long non-coding RNA (lncRNA) loci (Liu et al., 2017). This study considerably increased the number of known functional lncRNAs essential for cell growth and highlighted the strong specificity of lncRNA functions in different cell types. Therefore, cell type-specific complexities in the human non-coding genome may have important implications for normal biology and disease states, which should be considered in both the investigation of pathophysiological mechanisms and development of therapeutic approaches.

Great efforts are currently directed toward improvements of CRISPR-based editing, in particular, to increase Cas9 efficiency that arises from random nucleotide insertions or deletions after DSBs formation and to reduce off-target effects (Miyaoka et al., 2016). Increased precision of CRISPR-Cas9 technology holds great promises for future clinical translation. Base editors (BEs), for the correction of missense mutations commonly associated with diseases have recently been developed as an alternative to the conventional system. BEs enable editing of specific bases without the induction of DSBs, and they make use of dCas9 or a nickase version of Cas9 (nCas9) for the generation of single-filament breaks fused to a deaminase enzyme (Komor et al., 2016; Gaudelli et al., 2017; Rees and Liu, 2018). More recently, a modification based on a nCas9-reverse transcriptase construct, prime editor (PE), has been developed (Anzalone et al., 2019). PEs have the potential to offer more flexibility, interconverting any nucleotide or producing small insertions and deletions, which are commonly reported as disease-causing variants in human diseases. Both BEs and PEs have been applied to generate and correct mutations in iPSCs (Sürün et al., 2020). The study showed that BE is more efficient than nuclease-based HDR in this cell type, and PE can successfully be applied in iPSCs for changing more nucleotides simultaneously. Although improvements in efficiency, specificity, and deliverability for BEs and PEs are needed for further therapeutic applications (Anzalone et al., 2020), these new tools offer an opportunity to further expand genome editing capabilities. However, cell-state and cell-type determinants are still limiting gene editing. Therefore, continuous improvement efforts will be crucial to ensure that these technologies can accomplish their full potential.

## The Challenges and Limitations of Genome Editing in Neuronal Disease Models

Although the synergy of genome editing and the iPSC system is a powerful one, there remain inherent challenges for both disease modeling and CRISPR screens.

These include several fundamental shortcomings of iPSC-derived cell models as well as issues specific to genome editing. As discussed, even after long periods in culture, iPSC-derived neurons only reflect late fetal developmental stages, as shown by recent advances in electrophysiological and transcriptomic profiling (Velasco et al., 2019; Logan et al., 2021). While this is acceptable for early-onset and/or highly penetrant monogenic disorders with cell-autonomous phenotypes, there remains concern that later-onset phenotypes or those where environmental factors are important may not be recapitulated *in vitro*. Approaches to mitigate this include chemical cues, progerin expression, telomere shortening, and direct differentiation where reprogramming is avoided (Ziff and Patani, 2019). The immanent variability and heterogeneity of iPSC-derived neurons are due to a number of factors, including reprogramming-induced epigenetic changes and genomic instability, genetic background differences, and differing propensity to differentiation (Soldner and Jaenisch, 2012). To add to this complexity, myriad differentiation protocols are in use with significant line to line and lab to lab variability, raising concerns about reproducibility (Volpato and Webber, 2020). The use of isogenic controls, robust quality control using genetic profiling and functional validation of differentiated cells and single-cell assays such as scRNA sequencing (scRNA-Seq) will address some of these issues. Lastly, an undeniable and obvious feature of such *in vitro* systems is that they do not represent intact nervous systems or, indeed, whole brains and therefore cannot model the complex behavioral, motor and other phenotypes of many neurological disorders.

The major challenge of genome editing in iPSC models is that of unintended cleavage by site-specific nucleases leading to off-target effects. These could lead to perturbations of cell survival or differentiation pathways, which may alter the *in vitro* phenotype, as well as modification of the disease-associated phenotype (Soldner and Jaenisch, 2012). In addition, off-target effects could lead to genetic alterations of isogenic controls, confounding phenotypic interpretation (Musunuru, 2013). The specificity of gene editing in iPSCs has improved due to advances in Cas9 protein engineering and prime editing (Geng et al., 2020). Screening for off-target effects with whole-genome sequencing is not standardized in the field but would lead to a better understanding and delineation of these effects. The efficiency of editing of iPSCs was an issue in earlier protocols; this has improved with the advances in CRISPR/Cas9 discussed above and with inducible Cas9 (Wang G. et al., 2017), drug selection for successful clones or the use of electroporation (Geng et al., 2020).

## The Limitations of Crispr Screens in iPSC Neuronal Models

GWAS studies reveal a growing list of genetic loci associated with disease, identified within and outside coding regions, where they are thought to affect the expression of one or more coding genes through chromatin accessibility, transcription, splicing, and mRNA stability regulation (Buniello et al., 2019). The processes by which these variants contribute to disease are often unknown or controversial, in particular for complex disorders influenced by a combination of multiple genetic and environmental risk factors (Gallagher and Chen-Plotkin, 2018). Mechanistic understanding of disease-linked variants is, therefore, an important prerequisite for the development of new therapeutic strategies. iPSCs technology and the generation of isogenic lines on which allelic effects of a risk variant can be directly compared definitely increases the detection of single variant effects. However, resolving the direct biological consequence(s) of the expanding list of neurological disease-associated candidate loci needs sensitive and reliable methods to screen for the functionality of large numbers of variants and which are scalable to hundreds of SNPs. The parallel analysis of a wide number of genetic alterations opens up the possibility to dissect both the contribution of multiple genetic risk factors to complex disorders and the molecular mechanisms by which different mutations at same genes or related loci can account for variable disease phenotypes, with important implications for therapeutic approaches.

Pooled screens for cell-autonomous phenotypes that can be monitored through survival or cell sorting for specific cellular functions followed by next-generation sequencing to determine sgRNA frequencies should be prioritized given that their scalability enables a genome-wide approach (Li et al., 2017; Tian et al., 2019; Black et al., 2020). Pooled screens are more and more frequently coupled to scRNA-Seq to provide complex and rich information on cellular processes affected by gene perturbation (Dixit et al., 2016; Replogle et al., 2020; Schraivogel et al., 2020). While high-throughput sequencing of the transcriptome has rapidly expanded the capability to characterize the effects of neurological disorders-associated variants, the sheer number of genetic loci outside coding regions associated with disease makes it difficult to functionally characterize and validate all predicted connections. Thus, the successful application of parallel high-throughput techniques such as CRISPR screens, scRNA-Seq, scChIP-Seq, and scATAC–Seq, could expand the possibility for large-scale mapping and investigation of the human genome, although these readouts are currently expensive to implement.

However, the complex morphology of neurons and glia can limit the application of pooled screens to phenotypes identified through optical readout. Moreover, the pooled setup does not allow the interrogation of non-cell-autonomous phenotypes, in which the relevant phenotype is not physically coupled to the cell in which a gene is perturbed, such as excitation of target cells and interactions between neurons and glia, among others. Screens for non-cell-autonomous or complex functional or morphological phenotypes typically require arrayed approaches that allow setting a multitude of different readouts, such as high-content imaging (Bolognin et al., 2019; Yamaguchi et al., 2020) measurements of electrophysiological activity (Daily et al., 2017), and various biochemical assays (Medda et al., 2016; Zink et al., 2020). However, arrayed screens necessitate automated platforms for high-throughput measurement of the phenotypes of interest, and handling, culturing and analysis of hundreds of plates, a major limiting factor for most academic laboratories with limited capacity. Thus, arrayed screens usually entail the selection of a focused set of genes for perturbation when complex phenotypes are analyzed (Heman-Ackah et al., 2016; Deneault et al., 2018; Kwart et al., 2019), restraining the identification of disease mechanisms and/or new therapeutic targets to already known disease-associated variants. New advances in automated platforms for currently labor intensive iPSCs culture and complex differentiation protocols, both in 2D and 3D (Daily et al., 2017; Dhingra et al., 2020; Renner et al., 2020; Woodruff et al., 2020; Boussaad et al., 2021), will help in ensuring reproducibility of cellular phenotypes and high-throughput readouts. Technological development will in the future improve our ability to perform such screens and increase the number of variants that can be screened still further, expanding our knowledge about brain physiology and mechanisms that drive brain disorders with implications for therapeutic interventions.

## Future Directions for Genome Editing in iPSC Neuronal Systems

**Figure 4:**
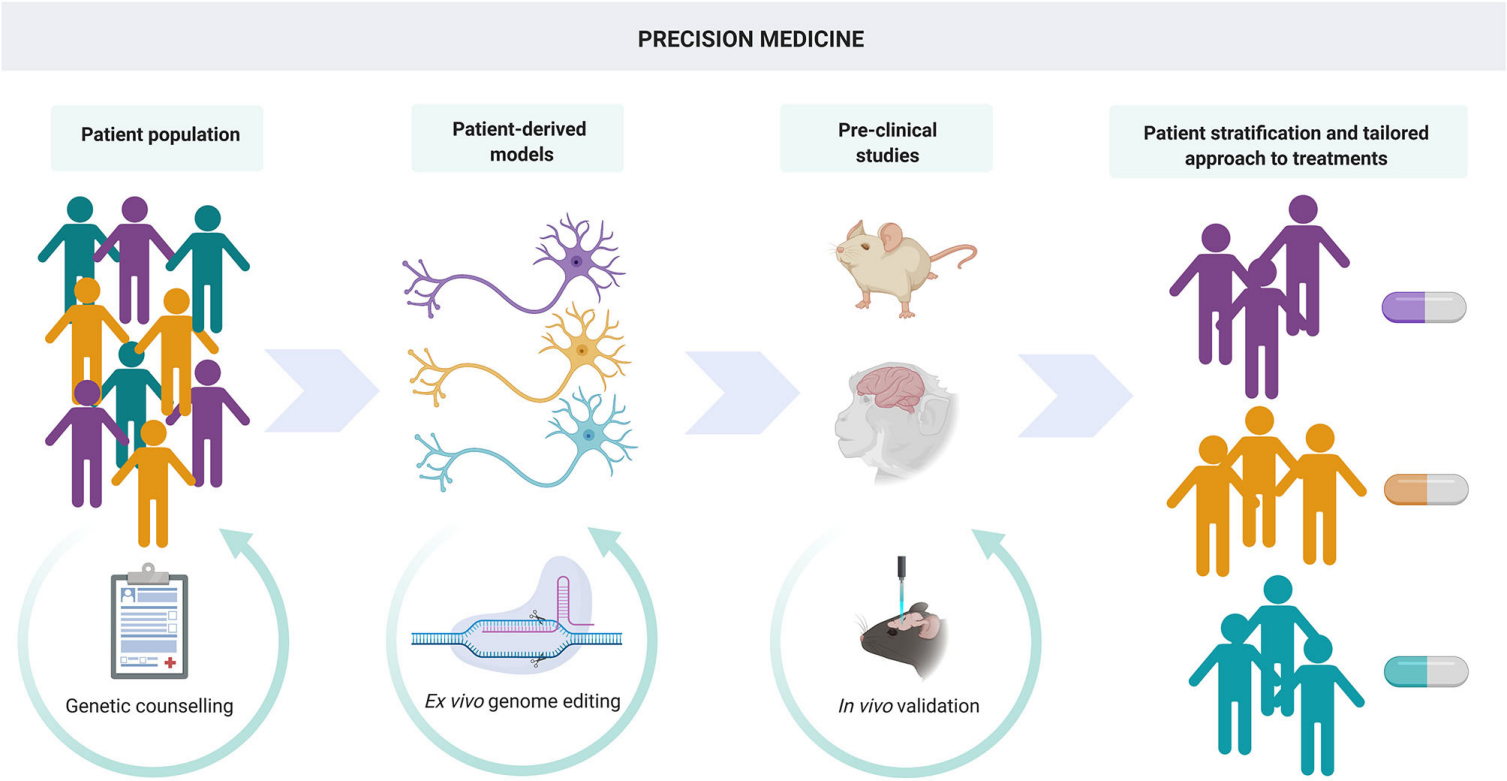
Precision medicine. iPSC and gene editing techniques can lead to advances not only in understanding disease mechanisms through *in vitro* modeling but also in the development of novel, personalized therapies. Genome editing of patient-derived cells in conjunction with assessment of efficacy and toxicity in *in vivo* models allows patient stratification and a tailored approach to treatment.

While the combination of genome engineering and iPSC-derived neuronal cells has led to many mechanistic discoveries, the driver for many researchers is the development of novel therapeutic approaches for these incurable disorders (Figure 4).

Many of these disease models (Supplementary Table 1) have acted as proof of principle of the rescue of the neuronal phenotype by gene editing. The knowledge gained from gene editing in iPSC systems is likely to add to research efforts to develop regenerative therapies for neurological disorders, currently most advanced in PD with a trial of transplantation of HLA-matched iPSC-derived dopaminergic neurons ongoing (Barker et al., 2017; Stoddard-Bennett and Pera, 2020). If this is successful, previous gene editing in iPSC PD models (Supplementary Table 1) could aid the development of edited patient-derived lines, which may overcome some issues of immunogenicity and would represent a truly personalized therapy. However, the most likely translation of insights gained in iPSC models will be toward *in vivo* CRISPR gene editing or promoter modulation. For example, iPSC-derived models of spinal muscular atrophy demonstrating rescue of the phenotype by CRISPR-mediated inclusion of exon 7 have paved the way toward the development of splicing modifiers, such as Risdiplam, which are now in clinical trials (Poirier et al., 2018). Similarly, in Duchenne muscular dystrophy, a variety of CRISPR-based methods have successfully restored dystrophin expression in iPSC models which was subsequently confirmed in murine and larger mammal models with current research focusing on clinical translation (Young et al., 2016; Mollanoori et al., 2020). iPSC models of Dravet syndrome (Supplementary Table 1), along with animal models, have contributed to understanding the cell-intrinsic disease mechanisms of *SCN1A* dysfunction. A recent study used CRISPRa to up-regulate *SCN1A* expression in interneurons in a Dravet mouse model, rescuing the tendency to febrile seizures (Colasante et al., 2020).

For translation into the clinic, a number of hurdles still exist for the majority of CRISPR-based therapeutic strategies. The first of these is delivery into the central nervous system with adequate transduction (with efficacy) of target brain regions and cell types. Viral delivery systems, largely AAV, have been used in mammal and humans to date, but concerns remain regarding immunogenicity. While AAVs are preferred to lentiviruses due to lack of genomic integration, their low packaging capacity is an issue due to the Cas endonuclease size. Recent advances in lipid or polymer-based nanoparticles allowing non-viral delivery hold promise for *in vivo* therapy (Wilbie et al., 2019). For example, gold nanoparticles complexed with donor DNA, Cas9 RNP, and the endosomal disruptive polymer PAsp(DET) rescued dystrophin expression and the muscle phenotype when delivered *in vivo* to a mouse model of Duchenne muscular dystrophy (Lee et al., 2017). The host immune response to the bacterial Cas protein and the presence of pre-existing immunity due to microbiome exposure is also a current barrier to translation. However, the largest hurdle for CRISPR-based therapies is the risk of off-target events occurring in the host genome. Better fidelity of gene editing as achieved by recent advances in CRISPR/Cas9 machinery will reduce this risk. The use of inducible Cas9 or CRISPR inhibitors such as small molecules or delivery of Cas9 as a ribonucleoprotein (RNP), rather than plasmid or viral vectors, can reduce the duration of editing and thus the risk of unwanted edits (Wilbie et al., 2019).

iPSC models coupled with gene editing are uniquely suited to help overcome these barriers. One of the major advantages of iPSC models is that they represent a human platform, which, combined with gene editing, offers the ability to model many different mutations in the same model system, as opposed to laborious generation of transgenic animal models. This means it is possible to create a personalized system in which to accurately test new therapies and aim to predict clinical response *in vitro* (Figure 4). For example, the starting iPSC clone can be compared to gene-edited clones to rigorously assess for off-target effects with transcriptomics. Recent developments in iPSC modeling, including 3D systems and the generation of assembloids, such as fusion of pallial and forebrain organoids (Sloan et al., 2018), are likely to better recapitulate neuronal interactions and networks and therefore will represent valuable readouts for assessing the impact of gene editing. This will also offer the opportunity to test delivery and targeting into specific cell or tissue types depending on promoter or nanoparticle delivery. In addition, neurons can be integrated with non-neuronal cell populations such as microglia (Marton and Paşca, 2020), which could be integral in addressing concerns regarding the immunogenicity potential of CRISPR and AAV systems. The ability to differentiate iPSCs into cells of any lineage could enable personalized off-target and toxicity testing in different organ systems, particularly if systemic administration is envisaged or in multi-system disorders to assess impact in different tissues. The emerging technology of personalized “organ on a chip” systems combined with high throughput transcriptomics is an exciting development that may have great relevance for testing and translation of precision therapies (Ronaldson-Bouchard and Vunjak-Novakovic, 2018; Ramme et al., 2019). Although iPSC-derived systems will not replace animal models for assessing future genetic treatments' safety, inter-species differences in sequence homology will impact targeting strategies and render testing in human-derived models an important part of the translational process (Wang et al., 2021).

In conclusion, genome engineering has transformed iPSC-based disease modeling for both Mendelian and more complex neurological disorders. The increasing accuracy of CRISPR gene editing, promoter modulation, and epigenome editing coupled with personalized, patient-derived iPSC model systems now has the potential for a paradigm shift in our understanding and treatment of neurological disorders.

## Author Contributions

AM, GR, AF, SB, and MK conceptual design and writing of the manuscript. All authors contributed to the article and approved the submitted version.

## Conflict of Interest

The authors declare that the research was conducted in the absence of any commercial or financial relationships that could be construed as a potential conflict of interest.
