# Supplementary material for: Genome Editing in iPSC-Based Neural Systems: From Disease Models to Future Therapeutic Strategies
Source: Front Genome Ed. 2021 Mar 15;3:630600. doi: 10.3389/fgeed.2021.630600 (PMC8525405; doi:10.3389/fgeed.2021.630600)
Supplement: Supplementary file 1 [file Table_1.docx]

**Table 1**: **Selected examples of iPSC-derived neurological disease models utilising gene editing techniques**. BBB blood brain barrier, Cas9 CRISPR associated protein 9, CRISPRa CRISPR activation, CRISPRi CRISPR inhibition, dCas9 deactivated Cas9, dCas9Tet1 dCas9 with Ten-Eleven Translocation enzyme (demethylation enzyme), GSK3 glycogen synthase kinase 3, eQTL expression quantitative trait loci, iPSC induced pluripotent stem cell, LOF loss of function, PM post-mortem, sgRNA single guide RNA, TALENs Transcription activator-like effector nucleases, ZFNs zinc finger nucleases.

| **Disorder** | **Gene** | **Differentiated cell type** | **Role of gene editing** | **Phenotypic insight** | **Reference** |
| --- | --- | --- | --- | --- | --- |
| **Neuromuscular disorders** | | | | | |
| Duchenne muscular dystrophy | *DMD* | Myotubes | CRISPR/Cas9 to delete five miRNA binding sites within the UTRN 3′ UTR leading to higher expression of endogenous utrophin | Editing resulted in 2x increased level of utrophin and dystrophin glycoprotein complex (DGC) restoration. This may represent a valid therapeutic approach regardless of dystrophin mutation type. | (Sengupta et al 2020) |
|  | *DMD* | Skeletal muscle cells | Correction of reading frame in patient-derived iPSCs with deletion of exon 44 using exon skipping, frameshifting and exon knock-in. | CRISPR-mediated exon 44 knock in restored full length dystrophin expression. Off-target mutagenesis analysis of edited cells compared to starting clone | (Li et al., 2015a) |
|  | *DMD* | Cardiomyocytes  Skeletal muscle cells | Correction of reading frame in patient-derived iPSCs with deletion of exons 48-50 by both reframing and exon skipping approaches | Editing of patient-derived cardiomyocytes rescued abnormal mitochondrial respiratory capacity | (Zhang et al., 2017) |
|  | *DMD* | Cardiomyocytes  Skeletal muscle myotubes | Restoration of reading frame in patient-derived cardiomyocytes and skeletal muscle with exon 45-55 deletions | Frame-restored myocytes and cardiomyocytes showed rescue of dystrophin expression, hypoosmotic stress response and miR31 to Becker muscular dystrophy levels. Edited myocytes engrafted and restored the dystrophin glycoprotein complex in muscular dystrophy mice. | (Young et al., 2016) |
| Spinal Muscular Atrophy (SMA) | *SMN1* | Motor neurons (MNs) | Gene conversion of SMN2 to a SMN1-like gene by inclusion of exon 7 using CRISPR/Cpf1 with single stranded oligodeoxynucletides (ssODN). | Gene editing corrected SMN expression, number of sub-nuclear bodies, MN survival, NMJ formation and expression of vesicular acetylcholine transporter. Off-target analysis in iPSCs showed correction to be efficient. | (Zhou et al., 2018) |
|  | *SMN1* | Mesenchymal stem cells (MSCs) | SMN1 targeted into the ribosomal DNA locus of SMA patient-derived iPSCs using rDNa-targeting vector and TALEN nickase | Gene editing restored SMN1 protein expression and number of sub-nuclear bodies. | (Feng et al., 2018) |
| **Dementia** | | | | | |
| Alzheimer disease (AD) | *PSEN1* | Cerebral organoids | CRISPR/Cas9 used to generate isogenic controls | AD organoids had increased Aβ42/Aβ40 ratio, abnormal calcium transients, and neuronal hyperactivity on calcium imaging compared to corrected controls. | (Yin, 2021) |
|  | *APP* and *PSEN1* | Cortical neurons and cerebral organoids | CRISPR/Cas9 used to generate isogenic controls | Hyperexcitability is seen in AD brains and mouse models and correlates with cognitive decline but basis unclear. Increased excitatory bursting seen in AD neurons and organoids compared to controls, possibly due to decrease in neurite length. Increased sodium current density and increased excitatory and decreased inhibitory synaptic activity also noted. This was treatable by α-secretase inhibitors, suggesting abnormal APP processing may be contributing. | (Ghatak et al., 2019) |
|  | Sporadic AD*/APOE4* | Cortical neurons and cerebral organoids | ﻿A homozygous APOE3 (low risk of AD) iPSC line from a normal aged donor was edited with CRISPR/Cas9 to APOE4 (high risk of AD). A APOE4 homozygous iPSC line from a patient with late-onset AD was edited to APOE3. | Using gene editing, a model of sporadic late onset AD was created. Sporadic AD neurons showed accelerated neuronal differentiation and reduced renewal of neural progenitors. Transcriptomic analysis revealed abnormal function of REST, a transcriptional repressor and central regulator of neuronal differentiation. This suggests epigenetic dysregulation of neural gene networks is an early feature of sporadic AD. | (Meyer et al., 2019) |
|  | *APOE4, PSEN1, APP* | Induced microglial cells (iMGLs) | Generation of isogenic controls | The APOE4 sporadic AD genotype conferred abnormal phagocytosis and migration and exaggerated inflammatory response, whereas familial AD genes did not affect microglia to the same degree. This suggests differing disease mechanisms for familial and sporadic AD. | (Konttinen et al., 2019) |
|  | *APP and PSEN1* | Cortical neurons | CRISPR/Cas9 to create a panel of *APP* and *PSEN1* mutant lines from control lines, with corresponding isogenic controls. | Global transcriptomic analysis revealed dysregulation in overlapping networks between different genetic causes of familial AD, and implicated endocytosis-associated genes. | (Kwart et al., 2019) |
|  | *APP* and *PSEN1* | Cortical neurons | Knock-in of homozygous and heterozygous early onset AD variants to control lines, generating isogenic pairs | ﻿Aβ levels and secreted Aβ42:40 ratio correlated with mutation load in comparison to isogenic controls. | (Paquet et al., 2016) |
|  | *PSEN2* | Basal forebrain cholinergic neurons (BFCNs) | CRISPR-Cas9 mediated correction of PSEN2 mutation to generate isogenic control | The cholinergic hypothesis of AD is based on PM data but mechanisms are unclear. BFCNs with PSEN2 mutation showed increased Aβ42/40 and fewer spikes on current injection. Correction of the mutation in isogenic lines rescued both phenotypic features. | (Ortiz-Virumbrales et al., 2017) |
|  | *PSEN1* | Brain endothelial cells (iBECs) | CRISPR-Cas9 mediated correction of *PSEN1* mutation to generate isogenic control | BBB may be disrupted in AD affecting delivery of drugs and novel therapies. AD iBECS had altered tight and adherin junction protein expression. AD and isogenic iBECs responded differently to focused ultrasound and microbubbles as a method to open the BBB. | (Oikari et al., 2020) |
|  | *APOE4* | Cortical neurons, astrocytes , microglial-like cells and cerebral organoids | CRISPR-Cas9 to create isogenic iPSCs homozygous for APOE3 from sporadic AD patient iPSCs | Conversion of *APOE4* genotype in sporadic AD patient lines to *APOE3* was sufficient to reverse the majority of AD-related phenotypes observed in multiple cell types. These included altered transcriptional profile, inability of glial cells to clear extracellular Aβ and increased Aβ aggregates in organoids. This suggests *APOE4* mediates AD pathology via impaired astrocyte and microglia mediated Aβ clearance. | (Lin et al., 2018) |
| Frontotemporal dementia (FTD) | *﻿CHMP2B* | Cortical neurons and astrocytes | CRISPR/Cas9 genome editing to generate corrected isogenic controls | Hypometabolism of glucose on PET is a biomarker of disease progression but metabolic landscape of FTD not understood. A number of comparative metabolic assays in mutant and repaired neurons revealed dysregulation of glutamate-glutamine homeostasis, potentially a target for future therapies. | (Aldana et al. 2020) |
|  | *MAPT* | Cerebral organoids dissociated to 2D cortical neurons | CRISPR/Cas9 genome editing to generate corrected isogenic controls and homozygous *MAPT* mutant lines from heterozygous patient lines | *MAPT* mutant neurons showed mislocalised Tau protein with reduced phosphorylation compared to isogenic controls, which may represent an early step in events leading to neurodegeneration. *MAPT* mutant neurons also showed axonal degeneration compared to isogenic controls which was partially rescued by a microtubule stabiliser, suggesting altered microtubule dynamics. | (Nakamura et al., 2019) |
|  | *MAPT* | Cortical neurons | ZFNs to introduce *MAPT* mutations into healthy donor iPSCs. | Whole transcriptome analysis revealed that a *MAPT* mutation drove differences in neuronal subtypes in vitro and was linked to aberrant WNT signaling. This was seen in both gene-edited and patient-derived lines. | (Verheyen et al., 2018) |
|  | *MAPT* | NG2 induced excitatory neurons | CRISPR/Cas9 genome editing to generate corrected isogenic controls and to introduce mutation into healthy controls | A novel role for the microtubule protein EB3 which accumulated in the axon initial segment of mutant but not corrected neurons, leading to loss of activity-dependent plasticity and abnormal neuronal network function | (Sohn et al., 2019) |
| **Movement disorders** | | | | | |
| Friedrich’s ataxia | *FXN* | Dorsal root ganglion(DRG) organoid-derived sensory neurons | CRISPR-Cas9 excision of either expanded GAA repeats or entire intron 1 | DRG-derived sensory neurons were co-cultured with intrafusal muscle fibres. Excision of intron 1 rather than just the expanded GAA tract led to restoration of frataxin expression, axonal growth deficit and mitochondrial dysfunction in patient-derived neurons compared to control levels. | (Mazzara et al., 2020) |
| Friedrich’s ataxia | *FXN* | Cortical neurons | ZFN excision of expanded GAA repeat in patient-derived iPSC lines | Gene editing resulted in increased frataxin expression in patient-derived lymphoblasts, fibroblasts and increased aconitase activity and ATP in iPSC-derived neurons | (Li et al., 2015b) |
| HSP | *SPG11* | Cortical neurons | CRISPR knockout of *SPG11* in control lines | Tidesglusib (GSK3 inhibitor) fully rescued the phenotype in patient-derived neurons but did not rescue apoptosis in edited control lines. | (Pozner et al., 2018) |
| Huntington disease | *HTT* | - | CRISPR-Cas9 mediated editing of abnormal CAG repeats in patient-derived line to generate isogenic controls. CRISPR was also used to generate a line with complete knockout of *HTT* | Several previous studies have used extremely long CAG repeat tracts not reflective of CAG repeat length in patients. | (Dabrowska et al., 2020) |
|  | *HTT* | NPCs, cortical neurons, myotubes and hepatocytes | TALENs used to engineer CAG repeats of varying length in control human embryonic stem cell lines | Cell-type specific phenotypes including mitochondrial dysfunction were related to CAG repeat length | (Ooi et al, 2019) |
|  | *HTT* | Cortical neurons and medium spiny neurons | ﻿CRISPR/Cas9 and piggyBac-based gene-editing to correct the expanded CAG in patient-derived lines. | Impaired neural rosette formation and defects in mitochondrial function in HTT neurons were rescued by gene editing. In gene expression studies, apparent differences between HTT and control lines were absent between HTT and corrected lines, therefore this was not an HTT-dependent effect. | (Xu et al., 2017) |
|  | *HTT* | Striatal neurons | CRISPR-Cas9 mediated excision of abnormal CAG repeats in patient-derived iPSCs to generate isogenic lines. | Reduction of CAG repeats to 21 rescued cell death and abnormal mitochondrial respiration. Gene expression differences in cadherin and TGF-β pathways were also rescued in isogenic lines. The differences between control and HTT neurons were many times greater than those between isogenic and HTT neurons. | (An et al 2012) |
| Myotonic dystrophy | *DMPK* | Mesangioblast-like cells | CRISPR-Cas9 mediated excision of abnormal CTG repeats | Editing rescued nuclear accumulation of ribonuclear foci, abnormal sequestration of the RNA-binding protein MBNL1 and aberrant splicing patterns. Although the excision was not allele-specific, excision of CTG repeats in the WT allele did not affect DMPK mRNA levels. This could represent a proof of principle for use of CRISPR excision in other repeat expansion disorders. | (Dastidar et al., 2018) |
| Parkinson disease | *LRRK2* | Midbrain dopaminergic neurons | CRISPR/Cas9 genome editing of patient-derived iPSCs to generate corrected isogenic controls | *LRKK2* shown to impair calcium homeostasis with an effect on axonal growth and integrity in mutant as compared to isogenic lines | (Korecka et al., 2019) |
|  | *LRRK2* | Neuroepithelial stem cells | CRISPR/Cas9 genome editing to generate corrected isogenic controls | Isogenic controls discerned *LRKK2* dependent and independent cellular phenotypes. Also able to screen for other phenotypes such as abnormal serine metabolism. Phenotypes were only partly *LRKK2* dependent, indicating an effect of genetic background in keeping with incomplete penetrance and variable phenotype. Identified *SRR* as a possible susceptibility factor. | (Nickels et al., 2019) |
|  | *PARK2* | Dopaminergic neurons | CRISPR knockout of *PARK2* in control lines with paired isogenic control | Gene expression and genome wide DNA methylation assay revealed increase in COMT with DNA hypomethylation. Increased COMT at presynaptic nerve terminals could metabolise dopamine and alter dopaminergic neurotransmission. This suggests that COMT inhibitors may be helpful in PD. | (Kuzumaki et al., 2019) |
|  | *PARK2* | Dopaminergic neurons | CRISPR knockout of *PARK2* in control lines with paired isogenic control | Mass spectrometry based proteomics on *PARK2* KO neurons and isogenic controls revealed specific effect of parkin dysfunction including oxidative stress, mitochondrial respiration and morphology and cell viability with decreased survival in *PARK2* KO neurons. | (Bogetofte et al., 2019) |
|  | *LRRK2* | Midbrain organoids and dopaminergic neurons | CRISPR/Cas9 genome editing to introduce *LRRK2* mutation into control iPSCs. | Mutant organoids recapitulated the human PD phenotype with abnormal localization of αsynuclein, which was not seen in isogenic controls. Transcriptomic analysis identified *TXNIP* as a novel regulator of αsynuclein in organoids but not 2D cultures. *TXNIP* knockdown improved αsynuclein accumulation. | (Kim et al., 2019) |
|  | *SNCA* |  | ﻿dCas9 fused with DMNT3A to demethylate *SNCA* in patient-derived iPSCs | DNA methylation is known to affect *SNCA* transcription and PD post-mortem brains sow altered methylation profiles. Dopaminergic neurons differentiated from edited iPSCs showed rescue of *SNCA* over-expression, αsynuclein levels and mitophagy defects without altering the overall cell methylome. | (Kantor et al., 2018) |
|  | *PARK2* | Induced neurons (iNs) | CRISPR to knock-out *THAP11* from control iPSCs | CRISPR screen in HEK293 cells identified *THAP11* as regulator of the PARKIN-mediated mitophagy. Knockout of *THAP11* in an isogenic pair confirmed the effect on mitophagy. | (Potting et al., 2017) |
|  | *SNCA* | - | ZFNs used to introduce mutations into control iPSCs and to correct mutations in patient-derived lines. | Validation of gene editing methodology and generation of isogenic pairs. | (Soldner et al., 2011) |
|  | *LRRK2* | Midbrain dopaminergic neurons | ZFN correction of *LRRK2* patient lines and knock-in of *LRRK2* to healthy control line. | Correction of *LRRK2* mutation rescued neurite outgrowth, abnormal autophagy and tau and αsynuclein accumulation. Transcriptomic comparison of isogenic pairs allowed identification of abnormal ERK-dependent phosphorylation. ERK inhibition improved many of the PD cellular phenotypes. | (Reinhardt et al., 2013) |
| Alternating hemiplegia of childhood | *ATP1A3* | Cortical glutamatergic and GABAergic neurons | CRISPR to correct mutation in patient-derived iPSCs | Mutant lines had upregulated *ATP1A3* transcripts compared to isogenic controls. A hyperactivity phenotype in MEA analysis was seen following heat stress which did not respond to flunarizine. | (Snow et al., 2020) |
| **Other neurodegenerative disorders** | | | | | |
| Amyotrophic lateral sclerosis | *C9orf72* | Motor neurons (MNs) | ﻿CRISPR/Cas9 replacement of excised region with a donor template carrying the wild-type repeat size | Correction restored normal gene expression and methylation of *C9orf72*. RNAseq of mutant versus isogenic control identified enrichment of ALS-relevant pathways and potential new targets including synaptotagmin11. | (Ababneh et al., 2020) |
|  | *FUS* | Motor neurons (MNs) | CRISPR correction of ALS patient lines and knock-in of ALS mutations to control lines. | Energy metabolism previously implicated in ALS. Metabolic testing showed a lactate oxidative metabolic switch during differentiation but this was also seen in isogenic controls and therefore likely a normal feature of neuronal differentiation. This suggested that metabolic dysfunction is not the cause of ALS phenotypes in iPSC-derived MNs. | (Vandoorne et al.) |
|  | *C9orf72, FUS, SOD1, TDP43* | Motor neurons | CRISPR/Cas9 genome editing to generate corrected isogenic controls | Mutation-specific difference in calcium dynamics and glutamate receptor expression and function in ALS neurons. This has implications for current use of riluzole | (Bursch et al., 2019) |
|  | *FUS* | Motor neurons | TALEN and CRISPR genome editing to generate corrected isogenic patient-derived pair and to knock-in a mutation to a control line | Axonal damage is seen in ALS but the mechanisms are unclear. Imaging analysis and RNA profiling revealed aberrant branching in FUS-mutant MNs compared to isogenic controls with increased levels of FosB-mRNA. Suppression of FosB rescued the abnormal branching. | (Akiyama et al., 2019) |
|  | *CHCD10/CHCHD2* | Motor neurons | CRISPR knockout of either *CHCD10* or *CHCHD2* | Loss of either gene results in similar alteration in mitochondrial respiration which may underpin their role in PD and ALS. *CHCD10* and *CHCHD2* show overlapping transcriptome profiles with abnormalities in synaptic gene expression compared to isogenic control line | (Harjuhaahto et al., 2020) |
| Progressive supranuclear palsy | *MAPT* | iNeurons | CRISPR generation of corrected isogenic controls | Mutated line more sensitive to cell death induced by chromium and nickel than isogenic control line. Confirmed the theory that heavy metal exposure is a risk for tau-opathies. | (Alquezar et al., 2020) |
| **Epilepsy** | | | | | |
| Genetic epilepsy with febrile seizures+ (GEFS+) | *SCN1A* | Excitatory and inhibitory cortical neurons | CRISPR generation of corrected isogenic controls from a heterozygous patient mutant iPSC line and creation of a homozygous mutant from an unaffected sibling. | Mutant *SCN1A* lines showed impairment of action potential firing and sodium current density in inhibitory neurons, which was gene dose dependent, with the homozygous line most severely affected. There were electrophysiological differences between the control and patient-lines which were not *SCN1A*-dependent, indicating genetic background did play a role. | (Xie et al., 2020) |
| Familial focal epilepsy with febrile seizures | *SCN1A* | ﻿GABAergic neurons, glutamatergic neurons and glial cells | Generation of corrected isogenic controls from heterozygous patient mutant iPSC line using TALENs and fluorescent reporter knock-in using CRISPR to identify GABAergic neurons | *SCN1A* mutant GABAergic neurons showed impaired AP firing and reduced post synaptic inhibitory potentials, indicating a selective loss of function in GABAergic neurons, confirming findings from other studies which had lacked isogenic controls. | (Liu et al., 2016) |
| Dravet syndrome | *SCN1A* | Cardiomyocytes | CRISPR-Cas9 used to gene edit *SCN1A* deletion into control iPSCs for comparison with iPSCs from 4 patients | Both patient-derived *SCN1A* and knock-in control lines exhibited increased sodium current density and spontaneous contraction rates, partly explained by compensatory increase in *SCN5A* expression. Use of the gene edited control line showed that haploinsufficiency of *SCN1A* was sufficient to cause increase in sodium current. High risk of SUDEP may result from predisposition to cardiac arrhythmias | (Frasier et al., 2018) |
| Migrating partial seizures of infancy | *KCNT1* | Cortical neurons | Gene editing to create a homozygous *KCNT1* mutant line from a control iPSC line | *KCNT1* homozygous neurons showed increased sodium dependent potassium currents and increased network excitability in MEA compared to wild-type isogenic controls. This study suggested that the disease mechanisms may be related to hypersynchronicity in neuronal networks. | (Quraishi et al., 2019) |
| Tuberous sclerosis (TS) | *TSC1/2* | Cerebellar Purkinje cells | CRISPR correction of *TSC2* mutation to generate isogenic controls and TALEN-engineered biallelic patient lines to mimic TSC tubers. | mTOR activation, defects in differentiation and RNA regulation and reduced synaptic activity were seen in mutant versus isogenic controls and the effect was gene dose dependent. | (Sundberg et al., 2018) |
|  | *TSC2* | Cortical neurons | TALEN to induce frameshift in second allele of *TSC2* generating a biallelic mutant line. | Heterozygous mutations show mTOR hyperactivation but only biallelic mutations showed the hyperactivity and transcriptional dysregulation seen in cortical tubers, suggesting loss of heterozygosity may be required for tuber formation | (Winden et al., 2019) |
|  | *TSC1* | Neural progenitor cells | CRISPR to correct mutation in patient-derived line and to induce second hit to create biallelic line | Editing restored abnormal proliferation and neurite outgrowth. Rapamycin did not rescue the mutant lines to the same degree as gene editing, indicating early neurodevelopmental phenotypes may not be solely mTORC1 dependent | (Martin et al., 2020) |
|  | *TSC2* | Neural precursor cells (NPCs) and neural crest cells (NCCs) | CRISPR to induce inactivating mutations in *TSC2* in four control lines. | Identified lineage-specific differences in signaling mechanisms between NPCs and NCCs with relevance for use of MTOR and proteasome in TS associated tumours | (Delaney et al., 2020) |
|  | *TSC1/2* | 3D Cortical spheroids | Editing of *TSC1*/*TSC2* to generate homozygous and heterozygous LOF variants and a line with heterozygous *TSC2* LOF variant and an inducible LOF variant in the functional allele | Mosaic biallelic inactivation during expansion of neural progenitors leads to formation of dysplastic cells. MTORC1 hyperactivation led to increased glial lineage cells, in keeping with astrocytosis in cortical tubers. This study further confirmed the two hit hypothesis of tuber formation in TS patients. | (Blair et al., 2018) |
| **Autistic Spectrum Disorder** | | | | | |
|  | *AFF2/FMR2, ANOS1, ASTN2, ATRX, CACNA1C, CHD8, DLGAP2, KCNQ2, SCN2A, TENM1* | NGN2 induced neurons (iNs) | CRISPR mediated deletion of ASD risk genes in unaffected controls | RNA sequencing in KO lines revealed converging neuronal networks. Although each mutation had differing electrophysiological signatures, there was a common reduction in synaptic activity. | (Deneault et al., 2018) |
|  | *SHANK2* | Cortical neurons | CRISPR-Cas9 correction of patient-derived *SHANK2* lines and generation of homozygous KO from a control line. | Altered dendrite length, complexity, synapse number and connectivity were found in mutant neurons, phenocopied in homozygous gene edited control lines and rescued by gene correction in the isogenic control lines. | (Zaslavsky et al., 2019) |
|  | *CHD8* | Cerebral organoids | CRISPR-Cas9 knockout of *CHD8* in healthy control lines | RNAseq with comparison to isogenic controls showed that *CHD8* regulates the expression of other ASD genes such as *TCF4* and *AUT2* | (Wang et al., 2017) |
| **Other neurodevelopmental disorders** | | | | | |
| Fragile X syndrome | *FMR1* | Cortical neurons | CRISPR targeted deletion of CGG repeats using NHEJ | Removal of CGG repeats led to extensive demethylation of the upstream CpG island of the FMR1 promoter, an open chromatin state and initiation of transcription, clarifying the role of the CGG repeats in epigenetic regulation. Editing led to reactivation of FMR1 in iPSC-derived neurons to levels similar to those in control wild-type neurons. | (Park et al., 2015) |
|  | *FMR1* | Cortical neurons | Targeted demethylation of the CGG expansion using dCas9-Tet1/sgRNA | Epigenetic editing restored *FMR1* expression and rescued electrophysiological abnormalities. Demethylation was also effective in post-mitotic neurons. | (Liu et al., 2018) |
|  | *FMR1* | Cortical neurons | Isogenic controls generated by CRISPR mediated deletion of CGG repeat region from a patient-derived line. dCas9-Tet1 also used to reactivate *FMR1* in FXS neurons | Isogenic controls allowed confirmation of previous studies finding hyperactivity in FRAX neurons. Partial restoration of FMRP expression was sufficient to rescue hyperactivity in iPSC-derived neurons measured by MEA. This has implications for future therapies. | (Graef et al., 2020) |
|  | *FMR1* | Cortical neurons | CRISPR-Cas9 mediated deletion of entire *FMR1* gene in an embryonic stem cell line to generate an isogenic pair for comparison with patient-derived lines with differing lengths of CGG repeats. | FMRP deficient neurons show spontaneous action potentials at greater frequency but shorter duration. This could be attributed to loss of FMRP expression as both patient-derived neuronal lines were electrically identical to the CRISPR edited line lacking *FMR1* | (Das Sharma et al., 2020) |
| Rett Syndrome | *MECP2* | Cortical neurons | CRISPR-Cas9 correction of *MECP2* mutation in patient-derived line and knock-in of mutation to control iPSCs. | Proof of principle of efficient editing of *MECP2* with restoration of *MECP2* expression. | (Huong Le et al., 2019) |
|  | *MECP2* | Cortical neurons | CRISPR-Cas9 correction of *MECP2* mutation in patient-derived line | Use of two plasmid system resulted in a much improved efficiency at 80% with restoration of *MECP2* expression. | (Croci et al., 2020) |
| Cockayne syndrome | *ERCC6* | Mesenchymal stem cells (MScs) and neural stem cells(NSCs) | CRISPR-Cas9 correction of *ERCC6* mutation in patient-derived line | Mutant *ERCC6* MSCs showed increased susceptibility to DNA damage by UV irradiation, recapitulating the premature aging defects in vivo; this was rescued by CRISPR correction. Transcriptomic analysis revealed ﻿rescue of impaired DNA damage repair, chromatin disorganization, and compromised cell proliferation genes in corrected lines. | (Wang et al., 2020) |
| Angelman syndrome (AS) | *UBE3A* | Forebrain cortical neurons | CRISPR-Cas9 knock out of *UBE3A* in a control line | AS neurons showed impaired maturation of resting membrane potential and action potential firing, decreased synaptic activity and reduced synaptic plasticity. Knockout lines confirmed this effect was due to *UBE3A* loss of function. Activation of the paternal allele with topoisomerase inhibitor rescued the AS phenotype. | (Fink et al., 2017) |
| Down syndrome | Trisomy 21 | Cortical neurons | CRISPR used to delete the supernumerary copy of *APP* in T21 lines and inducible CRISPRa used to upregulate *APP* expression. | *APP* gene dosage in T21 is responsible for increased βamyloid production but not for other tau-related Alzheimers phenotypes or apoptosis, indicating other mechanisms at play. | (Ovchinnikov et al., 2018) |
| **Metabolic disorders with neurological features** | | | | | |
| Nieman-Pick type C disease | *NPC1* | Neurons  Hepatocytes | Paired isogenic controls generated using TALENs | Confirmation of role of *NPC1* in autophagy with rescue of disease phenotype. Screening for autophagy-inducing compounds | (Maetzel et al., 2014) |
| Neuronal ceroid lipofuscinosis (Batten disease) | *CLN3* | - | ﻿CRISPR-Cas9-based homology-dependent repair of deletion in *CLN3* | Gene editing of both homozygous and compound heterozygous mutations restored *CLN3* expression to wild-type levels. | (Burnight et al., 2018) |
| Sandhoff disease | *HEXB* | Cerebral organoids | ﻿CRISPR-Cas9-based correction of mutation in *HEXB* in patient-derived iPSC | *HEXB* organoids accumulated GM2 ganglioside, this was rescued by gene correction. Transcriptomic analysis revealed delayed neuronal maturation in *HEXB* organoids compared to isogenic controls, representing an early neurodevelopmental phenotype. | (Allende et al., 2018) |
| **Psychiatric disorders** | | | | | |
| Schizophrenia (SZ) | *FURIN* rs4702 and four top-ranked SZ eQTL genes *(FURIN, SNAP91, TSNARE1 and CLCN3)* | Neural precursors, induced NGN2 excitatory neurons, GABAergic neurons, astrocytes, cortical and subpallial spheroids | CRISPR-Cas9 editing of control lines to introduce the risk allele rs4702 in FURIN. CRISPRa/i was used to modulate expression of *SNAP91, TSNARE1* and *CLCN3*. | Common and rare SZ-associated variants were found in the same genes or involved in common pathways. These susceptibility variants converged on a collective synaptic defect. In addition, the effect sizes seen for susceptibility loci were larger than in PM brain studies. Altogether these results suggest common and rare schizophrenia risk variants synergise to a common defect in synaptic function. | (Schrode et al., 2019) |
| Schizophrenia | *DISC1* | Cortical neurons | TALEN to generate isogenic controls from patient-derived iPSCs and introduce mutations into a control line | Post-mortem studies have suggested abnormal kinase activity in schizophrenia. Kinome arrays profiled kinase activity and showed kinase dysregulation in *DISC1* mutant lines compared to isogenic controls. | (Bentea et al., 2019) |

Supplementary References

Ababneh, N. A., Scaber, J., Flynn, R., Douglas, A., Barbagallo, P., Candalija, A., et al. (2020). Correction of amyotrophic lateral sclerosis related phenotypes in induced pluripotent stem cell-derived motor neurons carrying a hexanucleotide expansion mutation in C9orf72 by CRISPR/Cas9 genome editing using homology-directed repair. *Hum. Mol. Genet.* 00, 1–18. doi:10.1093/hmg/ddaa106.

Aldana, B. I., Zhang, Y., Jensen, P., Chandrasekaran, A., Christensen, S. K., Nielsen, T. T., et al. (2020). Glutamate-glutamine homeostasis is perturbed in neurons and astrocytes derived from patient iPSC models of frontotemporal dementia. *Mol. Brain* 13, 125. doi:10.1186/s13041-020-00658-6.

Allende, M. L., Cook, E. K., Larman, B. C., Nugent, A., Brady, J. M., Golebiowski, D., et al. (2018). Cerebral organoids derived from Sandhoff disease-induced pluripotent stem cells exhibit impaired neurodifferentiation. *J. Lipid Res.* 59, 550–563. doi:10.1194/jlr.M081323.

An, M. C., Zhang, N., Scott, G., Montoro, D., Wittkop, T., Mooney, S., et al. (2012). Genetic Correction of Huntington’s Disease Phenotypes in Induced Pluripotent Stem Cells. *Cell Stem Cell* 11, 253–263. doi:10.1016/j.stem.2012.04.026.

Bentea, E., Depasquale, E. A. K., O’Donovan, S. M., Sullivan, C. R., Simmons, M., Meador-Woodruff, J. H., et al. (2019). Kinase network dysregulation in a human induced pluripotent stem cell model of DISC1 schizophrenia. *Mol. Omi.* 15, 173–188. doi:10.1039/c8mo00173a.

Bogetofte, H., Jensen, P., Ryding, M., Schmidt, S. I., Okarmus, J., Ritter, L., et al. (2019). PARK2 mutation causes metabolic disturbances and impaired survival of human iPSC-derived neurons. *Front. Cell. Neurosci.* 13, 1–14. doi:10.3389/fncel.2019.00297.

Burnight, E. R., Bohrer, L. R., Giacalone, J. C., Klaahsen, D. L., Daggett, H. T., East, J. S., et al. (2018). CRISPR-Cas9-Mediated Correction of the 1.02 kb Common Deletion in CLN3 in Induced Pluripotent Stem Cells from Patients with Batten Disease. *Cris. J.* 1, 75–87. doi:10.1089/crispr.2017.0015.

Bursch, F., Kalmbach, N., Naujock, M., Staege, S., Eggenschwiler, R., Abo-Rady, M., et al. (2019). Altered calcium dynamics and glutamate receptor properties in iPSC-derived motor neurons from ALS patients with C9orf72, FUS, SOD1 or TDP43 mutations. *Hum. Mol. Genet.* 28, 2835–2850. doi:10.1093/hmg/ddz107.

Croci, S., Carriero, M. L., Capitani, K., Daga, S., Donati, F., Frullanti, E., et al. (2020). High rate of HDR in gene editing of p.(Thr158Met) MECP2 mutational hotspot. *Eur. J. Hum. Genet.* 28, 1231–1242. doi:10.1038/s41431-020-0624-x.

Dabrowska, M., Ciolak, A., Kozlowska, E., Fiszer, A., and Olejniczak, M. (2020). Generation of new isogenic models of huntington’s disease using CRISPR-Cas9 technology. *Int. J. Mol. Sci.* 21. doi:10.3390/ijms21051854.

Das Sharma, S., Pal, R., Reddy, B. K., Selvaraj, B. T., Raj, N., Samaga, K. K., et al. (2020). Cortical neurons derived from human pluripotent stem cells lacking FMRP display altered spontaneous firing patterns. *Mol. Autism* 11, 52. doi:10.1186/s13229-020-00351-4.

Dastidar, S., Ardui, S., Singh, K., Majumdar, D., Nair, N., Fu, Y., et al. (2018). Efficient CRISPR/Cas9-mediated editing of trinucleotide repeat expansion in myotonic dystrophy patient-derived iPS and myogenic cells. *Nucleic Acids Res.* 46, 8275–8298. doi:10.1093/nar/gky548.

Feng, M., Liu, C., Xia, Y., Liu, B., Zhou, M., Li, Z., et al. (2018). Restoration of SMN expression in mesenchymal stem cells derived from gene-targeted patient-specific iPSCs. *J. Mol. Histol.* 49, 27–37. doi:10.1007/s10735-017-9744-1.

Fink, J. J., Robinson, T. M., Germain, N. D., Sirois, C. L., Bolduc, K. A., Ward, A. J., et al. (2017). Disrupted neuronal maturation in Angelman syndrome-derived induced pluripotent stem cells. *Nat. Commun.* 8. doi:10.1038/ncomms15038.

Ghatak, S., Dolatabadi, N., Trudler, D., Zhang, X., Wu, Y., Mohata, M., et al. (2019). Mechanisms of hyperexcitability in alzheimer’s disease hiPSC-derived neurons and cerebral organoids vs. Isogenic control. *Elife* 8, 1–22. doi:10.7554/eLife.50333.

Graef, J. D., Wu, H., Ng, C., Sun, C., Villegas, V., Qadir, D., et al. (2020). Partial FMRP expression is sufficient to normalize neuronal hyperactivity in Fragile X neurons. *Eur. J. Neurosci.* 51, 2143–2157. doi:10.1111/ejn.14660.

Harjuhaahto, S., Rasila, T. S., Molchanova, S. M., Woldegebriel, R., Kvist, J., Konovalova, S., et al. (2020). ALS and Parkinson’s disease genes CHCHD10 and CHCHD2 modify synaptic transcriptomes in human iPSC-derived motor neurons. *Neurobiol. Dis.* 141. doi:10.1016/j.nbd.2020.104940.

Huong Le, T. T., Tran, N. T., Lan Dao, T. M., Nguyen, D. D., Do, H. D., Ha, T. L., et al. (2019). Efficient and precise CRISPR/Cas9-mediated MECP2 modifications in human-induced pluripotent stem cells. *Front. Genet.* 10. doi:10.3389/fgene.2019.00625.

Kim, H., Park, H. J., Choi, H., Chang, Y., Park, H., Shin, J., et al. (2019). Modeling G2019S-LRRK2 Sporadic Parkinson’s Disease in 3D Midbrain Organoids. *Stem Cell Reports* 12, 518–531. doi:10.1016/j.stemcr.2019.01.020.

Kuzumaki, N., Suda, Y., Iwasawa, C., Narita, M., Sone, T., Watanabe, M., et al. (2019). Cell-specific overexpression of COMT in dopaminergic neurons of Parkinson’s disease. *Brain* 142, 1675–1689. doi:10.1093/brain/awz084.

Nakamura, M., Shiozawa, S., Tsuboi, D., Amano, M., Watanabe, H., Maeda, S., et al. (2019). Pathological Progression Induced by the Frontotemporal Dementia-Associated R406W Tau Mutation in Patient-Derived iPSCs. *Stem Cell Reports* 13, 684–699. doi:10.1016/j.stemcr.2019.08.011.

Ortiz-Virumbrales, M., Moreno, C. L., Kruglikov, I., Marazuela, P., Sproul, A., Jacob, S., et al. (2017). CRISPR/Cas9-Correctable mutation-related molecular and physiological phenotypes in iPSC-derived Alzheimer’s PSEN2N141I neurons. *Acta Neuropathol. Commun.* 5. doi:10.1186/s40478-017-0475-z.

Paquet, D., Kwart, D., Chen, A., Sproul, A., Jacob, S., Teo, S., et al. (2016). Efficient introduction of specific homozygous and heterozygous mutations using CRISPR/Cas9. doi:10.1038/nature17664.

Pozner, T., Schray, A., Regensburger, M., Lie, D. C., Schlötzer-Schrehardt, U., Winkler, J., et al. (2018). Tideglusib Rescues Neurite Pathology of SPG11 iPSC Derived Cortical Neurons. *Front. Neurosci.* 12. doi:10.3389/fnins.2018.00914.

Sengupta, K., Mishra, M. K., Loro, E., Spencer, M. J., Pyle, A. D., & Khurana, T. S. (2020). Genome Editing-Mediated Utrophin Upregulation in Duchenne Muscular Dystrophy Stem Cells. *Molecular therapy. Nucleic acids*, *22*, 500–509. doi:10.1016/j.omtn.2020.08.031.

Sohn, P. D., Huang, C. T. L., Yan, R., Fan, L., Tracy, T. E., Camargo, C. M., et al. (2019). Pathogenic Tau Impairs Axon Initial Segment Plasticity and Excitability Homeostasis. *Neuron* 104, 458-470.e5. doi:10.1016/j.neuron.2019.08.008.

Vandoorne, T., Veys, K., Guo, W., Sicart, A., Vints, K., Swijsen, A., et al. (2019). Differentiation but not ALS mutations in FUS rewires motor neuron metabolism. *Nat. Commun.* 10, 4147. doi:10.1038/s41467-019-12099-4.

Xie, Y., Ng, N. N., Safrina, O. S., Ramos, C. M., Ess, K. C., Schwartz, P. H., et al. (2020). Comparisons of dual isogenic human iPSC pairs identify functional alterations directly caused by an epilepsy associated SCN1A mutation. *Neurobiol. Dis.* 134. doi:10.1016/j.nbd.2019.104627.

Xu, X., Tay, Y., Sim, B., Yoon, S. I., Huang, Y., Ooi, J., et al. (2017). Reversal of Phenotypic Abnormalities by CRISPR/Cas9-Mediated Gene Correction in Huntington Disease Patient-Derived Induced Pluripotent Stem Cells. *Stem Cell Reports* 8, 619–633. doi:10.1016/j.stemcr.2017.01.022.

Yin, J., and Van Dongen, A. M. (2021) Enhanced Neuronal Activity and Asynchronous Calcium Transients Revealed in a 3D Organoid Model of Alzheimer's Disease. *ACS Biomater Sci Eng* 11;7(1):254-264. doi:10.1021/acsbiomaterials.0c01583.

Zaslavsky, K., Zhang, W. B., McCready, F. P., Rodrigues, D. C., Deneault, E., Loo, C., et al. (2019). SHANK2 mutations associated with autism spectrum disorder cause hyperconnectivity of human neurons. *Nat. Neurosci.* 22, 556–564. doi:10.1038/s41593-019-0365-8.

Zhang, Y., Long, C., Li, H., McAnally, J. R., Baskin, K. K., Shelton, J. M., et al. (2017). CRISPR-Cpf1 correction of muscular dystrophy mutations in human cardiomyocytes and mice. *Sci. Adv.* 3. doi:10.1126/sciadv.1602814.
